# Supplementary material for: An activity-specificity trade-off encoded in human transcription factors
Source: Nat Cell Biol. 2024 Jul 5;26(8):1309–21. doi: 10.1038/s41556-024-01411-0 (PMC11321997; doi:10.1038/s41556-024-01411-0)
Supplement: Supplementary file 1 — Supplementary note and supplementary figures. [file 41556_2024_1411_MOESM1_ESM.pdf]

nature cell biology

Supplementary information https://doi.org/10.1038/s41556-024-01411-0

An activity-specificity trade-off encoded in human transcription factors

In the format provided by the authors and unedited

Supplementary Information

An activity-specificity trade-off encoded in human transcription factors

Julian Naderi[1,2,8], Alexandre P. Magalhaes[1,8], Gözde Kibar[3], Gregoire Stik[4,7], Yaotian Zhang[1], Sebastian D. Mackowiak[1], Hannah M. Wieler[1], Francesca Rossi[1], Rene Buschow[5], Marie Christou-Kent[4], Marc Alcoverro-Bertran[4], Thomas Graf[4,6], Martin Vingron[3], Denes Hnisz[1]*

* Correspondence to: hnisz@molgen.mpg.de

CONTENTS

- Supplementary Note
- Supplementary Figures 1-4

Supplementary Note

Protein sequences and SLIMs

Aromatic residues are in bold face, Short linear motifs (L/F/Y/W XX L/F/Y/W) are underlined. The sequences are the translated protein sequences used in the *in vitro* droplet formation and transactivation assays. The SLiM counts are listed in **Table S3**.

HOXD4 IDR Wild type

MVMSS**Y**MVNSK**Y**VDPK**F**PPCEE**Y**LQGG**Y**LGEQGAD**YY**GGGAQGAD**F**QPPGL**Y**PRPD**F**GEQ
P**F**GGSGPGPGSALPARGHGQEPGGPGGH**Y**AAPGEPCPAPPAPPPAPLPGARA**Y**SQSDPKQP
PSGTALKQPAVV**Y**P**W**MKKV

HOXD4 IDR AroLITE A

MVMSSAMVNSKAVDPKAPPCEEALQGGALGEQGADAAGGGAQGADAQPPGLAPRPDAGEQ
PAGGSGPGPGSALPARGHGQEPGGPGGHAAAPGEPCPAPPAPPPAPLPGARAASQSDPKQP
PSGTALKQPAVVAPAMKKV

HOXD4 IDR AroLITE S

MVMSSSMVNSKSVDPKSPPCEESLQGGSLGEQGADSSGGGAQGADSQPPGLSPRPDSGEQ
PSGGSGPGPGSALPARGHGQEPGGPGGHSAAPGEPCPAPPAPPPAPLPGARASSQSDPKQP
PSGTALKQPAVVSPSMKKV

HOXD4 IDR AroLITE G

MVMSSGMVNSKGVDPKGPPCEEGLQGGGLGEQGADGGGGGAQGADGQPPGLGPRPDGG
EQPGGGSGPGPGSALPARGHGQEPGGPGGHGAAPGEPCPAPPAPPPAPLPGARAGSQSDP
KQPPSGTALKQPAVVGPGMKKV

HOXD4 IDR AroPLUS

MVMSS**Y**MVNSK**Y**VDPK**F**PPCEE**Y**LQGG**Y**LGEQGAD**YY**GGGAQGAD**F**QPPGL**Y**PRPD**F**GEQ
P**F**GGSGPG**Y**GSALPAR**Y**HGQEP**Y**GPGGH**Y**AAPGEPCP**Y**PPAPPP**Y**PLPGARA**Y**SQSDPK**Y**P
PSGTA**Y**KQPAVV**Y**P**W**MKKV

HOXD4 IDR AroPLUS LITE

MVMSS**Y**MVNSK**Y**VDPK**F**PPCEE**Y**LQGG**Y**LGEQGAD**YY**GGGAQGAD**F**QPPGL**Y**PRPD**F**GEQ
P**F**GGSGPGAGSALPARAHGQEPAGPGGH**Y**AAPGEPCPAPPAPPPAPLPGARA**Y**SQSDPKAPP
SGTAAKQPAVV**Y**P**W**MKKV

HOXD4 IDR AroPLUS patched
MVMSS**Y**MVNSK**Y**VDPK**FY**PPCEE**Y**LQGG**YY**LGEQGAD**YY**GGGAQGAD**F**QPPGL**YY**PRPD**F**
GEQP**FY**GGSGPGGSALPARHGQEPGPGGH**YY**AAPGEPCPPPAPPPPLPGARA**YY**SQSDPKP
PSGTAKQPAVV**YY**P**W**MKKV

HOXD4 IDR AroPLUS LITE patched
MVMSS**Y**MVNSK**Y**VDPK**F**APPCEE**Y**LQGG**Y**ALGEQGAD**YY**GGGAQGAD**F**QPPGL**Y**APRPD**F**
GEQP**F**AGGSGPGGSALPARHGQEPGPGGHA**Y**AAPGEPCPPPAPPPPLPGARA**Y**ASQSDPKP
PSGTAKQPAVVA**Y**P**W**MKKV

HOXD4 IDR AroPERFECT
MVMSSMVN**Y**SKVDPKPP**F**CEELQGGL**Y**GEQGADGG**Y**GAQGADQP**F**PGLPRPDG**F**EQPGGS
GP**F**GPGSALPA**Y**RGHGQEPG**Y**GPGGHAAP**Y**GEPCPAPP**Y**APPPAPLP**Y**GARASQSD**Y**PKQP
PSGT**Y**ALKQPAVV**W**PMKKV

HOXD4 IDR AroPERFECT -1
MVMSSMV**Y**NSKVDPKP**F**PCEELQGG**Y**LGEQGADG**Y**GGAQGADQ**F**PPGLPRPD**F**GEQPGGS
G**F**PGPGSALP**Y**ARGHGQEP**Y**GGPGGHAA**Y**PGEPCPAP**Y**PAPPPAPL**Y**PGARASQS**Y**DPKQP
PSG**Y**TALKQPAV**W**VPMKKV

HOXD4 IDR AroPERFECT -2
MVMSSM**Y**VNSKVDPK**F**PPCEELQG**Y**GLGEQGAD**Y**GGGAQGAD**F**QPPGLPRP**F**DGEQPGGS
FGPGPGSAL**Y**PARGHGQE**Y**PGGPGGHA**Y**APGEPCPA**Y**PPAPPPAP**Y**LPGARASQ**Y**SDPKQP
PS**Y**GTALKQPA**W**VVPMKKV

HOXD4 IDR Wild type YPWM(-)
MVMSS**Y**MVNSK**Y**VDPK**F**PPCEE**Y**LQGG**Y**LGEQGAD**YY**GGGAQGAD**F**QPPGL**Y**PRPD**F**GEQ
P**F**GGSGPGPGSALPARGHGQEPGGPGGH**Y**AAPGEPCPAPPAPPPAPLPGARA**Y**SQSDPKQP
PSGTALKQPAVVAPAMKKV

HOXD4 IDR AroLITE YPWM(+)

MVMSSAMVNSKAVDPKAPPCEEALQGGALGEQGADAAGGGAQGADAQPPGLAPRPDAGEQ
PAGGSGPGPGSALPARGHGQEPGGPGGHAAAPGEPCPAPPAPPPAPLPGARAASQSDPKQP
PSGTALKQPAVV**Y**P**W**MKKV

HOXD4 IDR AroPERFECT YPWM (+)

MVMSSMVN**Y**SKVDPKPP**F**CEELQGGL**Y**GEQGADGG**Y**GAQGADQP**F**PGLPRPDG**F**EQPGGS
GP**F**GPGSALPA**Y**RGHGQEPG**Y**GPGGHAAP**Y**GEPCPAPP**Y**APPPAPLP**Y**GARASQSD**Y**PKQP
PSGTALKQPAVV**Y**P**W**MKKV

HOXD4 wild type (N)

MVMSSMVNSKVDPK**F**PPCEE**Y**LQGG**Y**LGEQGAD**YY**GGGAQGAD**F**QPPGL**Y**PRPD**F**GEQP**F**
GGSGPGPGSAL

HOXD4 WT(N)-FUSNxs

MVMSSMVNSKVDPK**F**PPCEE**Y**LQGG**Y**LGEQGAD**YY**GGGAQGAD**F**QPPGL**Y**PRPD**F**GEQP**F**
GGSGPGPGSALASND**Y**TQQATQS**Y**GA**Y**PTQPGQG**Y**SQQSSQP**Y**GQQS**Y**SG**Y**SQSTDTSG**Y**
GQSS

FUSN

ASND**Y**TQQATQS**Y**GA**Y**PTQPGQG**Y**SQQSSQP**Y**GQQS**Y**SG**Y**SQSTDTSG**Y**GQSS**Y**SS**Y**GQS
QNTG**Y**GTQSTPQG**Y**GSTGG**Y**GSSQSSQSS**Y**GQQSS**Y**PG**Y**GQQPAPSSTSGS**Y**GSSSQSSS
YGQPQSGS**Y**SQQPS**Y**GGQQQS**Y**GQQQS**Y**NPPQG**Y**GQQNQ**Y**NSSSGGGGGGGGGN**Y**G
QDQSSMSSGGGSGGG**Y**GNQDQSGGGGSGG**Y**GQQDRGGRGRGGSGGGGGGGGGG**Y**NR
SSGG**Y**EPRGRGGGRGGRGGMGGSDRGG**F**NK**F**GGPRDQGSRHDSEQDNSDNNTI

FUSNxs

ASND**Y**TQQATQS**Y**GA**Y**PTQPGQG**Y**SQQSSQP**Y**GQQS**Y**SG**Y**SQSTDTSG**Y**GQSS

HOXC4 IDR Wild type

MIMSS**Y**LMDSN**Y**IDPK**F**PPCEE**Y**SQNS**Y**IPEHSPE**YY**GRTRESG**F**QHHHQEL**Y**PPPPPRPS**Y**P
ERQ**Y**SCTSLQGPGNSRGHGPAQAGHHHPEKSQSLCEPAPLSGASASPSPAPPACSQPAPDHP
SSAASKQPIV**Y**P**W**MKMSRGPYSIVSPKC

HOXC4 IDR AroLITE S

MIMSSALMDSNAIDPKAPPCEEASQNSAIPEHSPEAAGRTRESGAQHHHQELAPPPPPRPSAP
ERQASCTSLQGPGNSRGHGPAQAGHHHPEKSQSLCEPAPLSGASASPSPAPPACSQPAPDHP
SSAASKQPIVAPAMKMSRGPYSIVSPKC

HOXC4 IDR AroPERFECT

MIMSSLM**Y**DSNIDPKPPC**F**EESQNSIPEH**Y**SPEGRTRESG**F**QHHHQELPPP**Y**PPRPSPERQS**Y**
CTSLQGPGNS**Y**RGHGPAQAGH**Y**HHPEKSQSLC**Y**EPAPLSGASA**Y**SPSPAPPACS**Y**QPAPDHP
SSA**Y**ASKQPIVPM**W**KMSRGPYSIVSPKC

HOXB1 IDR Wild type

MD**Y**NRMNS**F**LE**Y**PLCNRGPSA**Y**SAHSAPTS**F**PPSSAQAVDS**Y**ASEGR**Y**GGGLSSPA**F**QQNS
G**Y**PAQQPPSTLGVP**F**PSSAPSG**Y**APAACSPS**Y**GPSQ**YY**PLGQSEGDGG**YF**HPSS**Y**GAQLGGL
SDG**Y**GAGGAGPGP**Y**PPQHPP**Y**GNEQTAS**F**APA**Y**ADLLSEDKETPCPSEPNTPTART**F**D**W**MKV
KRNPPKTAKVSEPGL

HOXB1 IDR AroLITE A

MDANRMNSALEAPLCNRGPSAASAHSAPTSAPPSSAQAVDSAASEGRAGGGLSSPAAQQNS
GAPAQQPPSTLGVPAPSSAPSGAAPAACSPSAGPSQAAPLGQSEGDGGAAHPSSAGAQLGG
LSDGAGAGGAGPGPAPPQHPPAGNEQTASAAPAAADLLSEDKETPCPSEPNTPTARTADAMK
VKRNPPKTAKVSEPGL

HOXB1 IDR AroPERFECT

MD**Y**NRMNSLE**Y**PLCNRGP**Y**SASAHSA**F**PTSPPSS**F**AQAVDSA**Y**SEGRGGG**Y**LSSPAQQ**F**NS
GPAQQ**Y**PPSTLGV**F**PPSSAPS**Y**GAPAACS**Y**PSGPSQP**Y**LGQSEGD**Y**GGHPSSG**F**AQLGGLS**Y**
DGGAGGA**Y**GPGPPPQ**Y**HPPGNEQ**Y**TASAPAA**F**DLLSEDK**Y**ETPCPSE**Y**PNTPTAR**F**TDMKVK
R**W**NPPKTAKVSEPGL

NANOG IDR Wild type

KQVKT**WF**QNQRMKSKR**W**QKNN**W**PKNSNGVTQKASAPT**Y**PSL**Y**SS**Y**HQGCLVNPTGNLPM**W**
SNQT**W**NNST**W**SNQTQNIQS**W**SNHS**W**NTQT**W**CTQS**W**NNQA**W**NSP**FY**NCGEESLQSCMQ**F**
QPNSPASDLEAAL

NANOG IDR AroLITE A

KQVKTAAQNQRMKSKRAQKNNAPKNSNGVTQKASAPTAPSLASSAHQGCLVNPTGNLPMAS
NQTANNSTASNQTQNIQSASNHSANTQTACTQSANNQAANSPAANCGEESLQSCMQAQPNSP
ASDLEAAL

EGR1 IDR Wild type
LRQKDKKADKSVVASSATSSLSS**Y**PSPVATS**Y**PSPVTTS**Y**PSPATTS**Y**PSPVPTS**F**SSPGSST**Y**
PSPVHSG**F**PSPSVATT**Y**SSVPPA**F**PAQVSS**F**PSSAVTNS**F**SASTGLSDMTAT**F**SPRTIEIC

EGR1 IDR AroLITE A
LRQKDKKADKSVVASSATSSLSSAPSPVATSAPSPVTTSAPSPATTSAPSPVPTSASSPGSSTA
PSPVHSGAPSPSVATTASSVPPAAPAQVSSAPSSAVTNSASASTGLSDMTATASPRTIEIC

EGR1 IDR AroSCRAMBLED
LRQKDKKADKSVVASSATSSLSS**Y**PSPVA**F**T**Y**SPSPVTTSPSP**Y**AT**Y**TSPSPVPTSSS**F**PGSS**Y**
FTPSPVHSGP**Y**SPSVATTSSVPPAPAQVSSPSSAV**F**TNS**F**SASTG**F**LSDMTATSPRTIEIC

EGR1 IDR AroPATCHY3
LRQKDKKADKSVVASSATSSLSS**YYYY**PSPVATSPSPVTTSPSPATTSPSPVPTSSSPGSSTPS
PVHSGPSPSVATT**YFFY**SSVPPAPAQVSSPSSAVTNS**FFFF**SASTGLSDMTATSPRTIEIC

EGR1 IDR AroPATCHY1
LRQKDKKADKSVVASSATSSLSSPSPVATSPSPVTTSPSPATTSPSPVPTSSSPGSST**YYYYFY**
YFFFFFPSPVHSGPSPSVATTSSVPPAPAQVSSPSSAVTNSSASTGLSDMTATSPRTIEIC

NFAT5 IDR Wild type
TMVKKEISSPARPCS**F**EEAMKAMKTTGCNLDKVNIIPNALMTPLIPSSMIKSEDVTPMEVTAEKR
SSTI**F**KTTKSVGSTQQTLENISNIAGNGS**F**SSPSSSHLPSENEKQQQIQPKA**Y**NPETLTTIQTQDI
SQPGT**F**PAVSASSQLPNSDALLQQATQ**F**QTRETQSREILQSDGTVVNLSQLTEASQQQQQSPL
QEQAQTLQQQISSNI**F**PSPNSVSQLQNTIQQLQAGS**F**TGSTASGSSGSVDLVQQVLEAQQQLS
SVL**F**SAPDGNENVQEQLSADI**F**QQVSQIQSGVSPGM**F**SSTEPTVHTRPDNLLPGRAESVHPQS
ENTLSNQQQQQQQQQQVMESSAAMVMEMQQSICQAAAQIQSEL**F**PSTASANGNLQQSPV**Y**Q
QTSHMMSALSTNEDMQMQCEL**F**SSPPAVSGNETSTTTTQQVATPGTTM**F**QTSSSGDGEETGT
QAKQIQNSV**F**QTMVQMQHSGDNQPQVNL**F**SSTKSMMSVQNSGTQQQGNGL**F**QQGNEMMSL
QSGN**F**LQQSSHSQAQL**F**HPQNPIADAQNLSQETQGSL**F**HSPNPIVHSQTSTTSSEQMQPPM**F**

HSQSTIAVLQGSSVPQDQQSTNI**F**LSQSPMNNLQTNTVAQEA**FF**AAPNSISPLQSTSNSEQQAA
FQQQAPISHIQTPMLSQEQAQPPQQGL**F**QPQVALGSLPPNPMPQSQQGTM**F**QSQHSIVAMQS
NSPSQEQQPPPPRRPLPPLPLQQSIL**F**SNQNTMATMASPKQPPPNMI**F**NPNQNPMANQEQQN
QSI**F**HQQSNMAPMNQEQQPMQ**F**QSQSTVSSLQNPGPTQSESSQTPL**F**HSSPQIQLVQGSPSS
QEQQVTL**F**LSPASMSALQTSINQQDMQQSPL**Y**SPQNNMPGIQGATSSPQPQATL**F**HNTAGGT
MNQLQNSPGSSQQTSGM**F**L**F**GIQNNCSQLLTSGPATLPDQLMAISQPGQPQNEGQPPVTTLLS
QQMPENSPLASSINTNQNIEKIDLLVSLQNQGNNLTGS**F**

NFAT5 IDR AroLITE A
TMVKKEISSPARPCSAEEAMKAMKTTGCNLDKVNIIPNALMTPLIPSSMIKSEDVTPMEVTAEKR
SSTIAKTTKSVGSTQQTLENISNIAGNGSASSPSSSHLPSENEKQQQIQPKAANPETLTTIQTQDI
SQPGTAPAVSASSQLPNSDALLQQATQAQTRETQSREILQSDGTVVNLSQLTEASQQQQQSPL
QEQAQTLQQQISSNIAPSPNSVSQLQNTIQQLQAGSATGSTASGSSGSVDLVQQVLEAQQQLS
SVLASAPDGNENVQEQLSADIAQQVSQIQSGVSPGMASSTEPTVHTRPDNLLPGRAESVHPQ
SENTLSNQQQQQQQQQVMESSAAMVMEMQQSICQAAAQIQSELAPSTASANGNLQQSPVA
QQTSHMMSALSTNEDMQMQCELASSPPAVSGNETSTTTTQQVATPGTTMAQTSSSGDGEET
GTQAKQIQNSVAQTMVQMQHSGDNQPQVNLASSTKSMMSVQNSGTQQQGNGLAQQGNEM
MSLQSGNALQQSSHSQAQLAHPQNPIADAQNLSQETQGSLAHSPNPIVHSQTSTTSSEQMQP
PMAHSQSTIAVLQGSSVPQDQQSTNIALSQSPMNNLQTNTVAQEAAAAAPNSISPLQSTSNSE
QQAAAQQQAPISHIQTPMLSQEQAQPPQQGLAQPQVALGSLPPNPMPQSQQGTMAQSQHSI
VAMQSNSPSQEQQQQQQQQQQSILASNQNTMATMASPKQPPPNMIANPNQNPMANQEQQ
NQSIAHQQSNMAPMNQEQQPMQAQSQSTVSSLQNPGPTQSESSQTPLAHSSPQIQLVQGSP
SSQEQQVTLALSPASMSALQTSINQQDMQQSPLASPQNNMPGIQGATSSPQPQATLAHNTAG
GTMNQLQNSPGSSQQTSGMALAGIQNNCSQLLTSGPATLPDQLMAISQPGQPQNEGQPPVTT
LLSQQMPENSPLASSINTNQNIEKIDLLVSLQNQGNNLTGSA

C/EBPα IDR Wild type
MRGRGRAGSPGGRRRRPAQAGGRRGSPCRENSNSPMESAD**FY**EAEPRPPMSSHLQSPPHA
PSSAA**F**G**F**PRGAGPAQPPAPPAAPEPLGGICEHETSIDISA**Y**IDPAA**F**NDE**F**LADL**F**QHSRQQEK
AKAAVGPTGGGGGGD**F**D**Y**PGAPAGPGGAVMPGGAHGPPPG**Y**GCAAAG**Y**LDGRLEPL**Y**ERV
GAPALRPLVIKQEPREEDEAKQLALAGL**F**P**Y**QPPPPPPPSHPHPHPPPAHLAAPHLQ**F**QIAHCG
Q

C/EBPα IDR AroLITE A

MRGRGRAGSPGGRRRRPAQAGGRRGSPCRENSNSPMESADAAEAEPRPPMSSHLQSPPHA
PSSAAAGAPRGAGPAQPPAPPAAPEPLGGICEHETSIDISAAIDPAAANDEALADLAQHSRQQE
KAKAAVGPTGGGGGGDADAPGAPAGPGGAVMPGGAHGPPPGAGCAAAGALDGRLEPLAER
VGAPALRPLVIKQEPREEDEAKQLALAGLAPAQPPPPPPPSHPHPHPPPAHLAAPHLQAQIAHC
GQ

C/EBPα IDR AroPERFECT IS15
MRG**F**RGRAGSPGGRRRRPA**Y**QAGGRRGSPCRENSN**F**SPMESADEAEPRPPM**F**SSHLQSPP
HAPSSAA**F**GPRGAGPAQPPAPPA**Y**APEPLGGICEHETSI**F**DISAIDPAANDELAD**F**LQHSRQQE
KAKAAVG**F**PTGGGGGGDDPGAPA**Y**GPGGAVMPGGAHGPP**Y**PGGCAAAGLDGRLEP**Y**LERV
GAPALRPLVIK**Y**QEPREEDEAKQLALA**F**GLPQPPPPPPPSHPH**Y**PHPPPAHLAAPHLQQ**F**IAHC
GQ

C/EBPα IDR AroPERFECT IS15 +1
MRGR**F**GRAGSPGGRRRRPAQ**Y**AGGRRGSPCRENSNS**F**PMESADEAEPRPPMS**F**SHLQSPP
HAPSSAAG**F**PRGAGPAQPPAPPAA**Y**PEPLGGICEHETSID**F**ISAIDPAANDELADL**F**QHSRQQE
KAKAAVGP**F**TGGGGGGDDPGAPAG**Y**PGGAVMPGGAHGPPP**Y**GGCAAAGLDGRLEPL**Y**ERV
GAPALRPLVIKQ**Y**EPREEDEAKQLALAG**F**LPQPPPPPPPSHPHP**Y**HPPPAHLAAPHLQQI**F**AHC
GQ

C/EBPα IDR AroPERFECT IS15 +2
MRGRG**F**RAGSPGGRRRRPAQA**Y**GGRRGSPCRENSNSP**F**MESADEAEPRPPMSS**F**HLQSPP
HAPSSAAGP**F**RGAGPAQPPAPPAAP**Y**EPLGGICEHETSIDI**F**SAIDPAANDELADLQ**F**HSRQQE
KAKAAVGPT**F**GGGGGGDDPGAPAGP**Y**GGAVMPGGAHGPPPG**Y**GCAAAGLDGRLEPLE**Y**RV
GAPALRPLVIKQE**Y**PREEDEAKQLALAGL**F**PQPPPPPPPSHPHPH**Y**PPPAHLAAPHLQQIA**F**HC
GQ

C/EBPα IDR AroPERFECT IS10
MRGRGRAGS**F**PGGRRRRPAQ**F**AGGRRGSPCR**Y**ENSNSPMESA**Y**DEAEPRPPMS**F**SHLQSP
PHAP**F**SSAAGPRGAG**F**PAQPPAPPAA**F**PEPLGGICEH**Y**ETSIDISAID**Y**PAANDELADL**F**QHSRQ
QEKAK**F**AAVGPTGGGG**F**GGDDPGAPAG**F**PGGAVMPGGA**F**HGPPPGGCAA**F**AGLDGRLEPL**F**
ERVGAPALRP**F**LVIKQEPREE**Y**DEAKQLALAG**Y**LPQPPPPPPP**Y**SHPHPHPPPA**Y**HLAAPHLQQ
I**Y**AHCGQ

C/EBPα IDR wild type (N)

MRGRGRAGSPGGRRRRPAQAGGRRGSPCRENSNSPMESAD**FY**EAEPRPPMSSHLQSPPHA
PSSAA**F**G**F**PRGAGPAQPPAPPAAPEPLGGICEHETSIDISA**Y**IDPAA**F**NDE**F**LADL**F**QHS

C/EBPα IDR WT(N)-IS15

MRGRGRAGSPGGRRRRPAQAGGRRGSPCRENSNSPMESAD**FY**EAEPRPPMSSHLQSPPHA
PSSAA**F**G**F**PRGAGPAQPPAPPAAPEPLGGICEHETSIDISA**Y**IDPAA**F**NDE**F**LADL**F**QHSRQQE
KAKAAVG**F**PTGGGGGGDDPGAPA**Y**GPGGAVMPGGAHGPP**Y**PGGCAAAGLDGRLEP**Y**LERV
GAPALRPLVIK**Y**QEPREEDEAKQLALA**F**GLPQPPPPPPPSHPH**Y**PHPPPAHLAAPHLQQ**F**IAHC
GQ

C/EBPα IDR WT(N)-FUSN

MRGRGRAGSPGGRRRRPAQAGGRRGSPCRENSNSPMESAD**FY**EAEPRPPMSSHLQSPPHA
PSSAA**F**G**F**PRGAGPAQPPAPPAAPEPLGGICEHETSIDISA**Y**IDPAA**F**NDE**F**LADL**F**QHSASND**Y**
TQQATQS**Y**GA**Y**PTQPGQG**Y**SQQSSQP**Y**GQQS**Y**SG**Y**SQSTDTSG**Y**GQSS**Y**SS**Y**GQSQNTG**Y**
GTQSTPQG**Y**GSTGG**Y**GSSQSSQSS**Y**GQQSS**Y**PG**Y**GQQPAPSSTSGS**Y**GSSSQSSS**Y**GQPQ
SGS**Y**SQQPS**Y**GGQQQS**Y**GQQQS**Y**NPPQG**Y**GQQNQ**Y**NSSSGGGGGGGGGGN**Y**GQDQSS
MSSGGGSGGG**Y**GNQDQSGGGGSGG**Y**GQQDRGGRGRGGSGGGGGGGGGG**Y**NRSSGG**Y**E
PRGRGGGRGGRGGMGGSDRGG**F**NK**F**GGPRDQGSRHDSEQDNSDNNTI

C/EBPα IDR WT(N)-FUSNxs

MRGRGRAGSPGGRRRRPAQAGGRRGSPCRENSNSPMESAD**FY**EAEPRPPMSSHLQSPPHA
PSSAA**F**G**F**PRGAGPAQPPAPPAAPEPLGGICEHETSIDISA**Y**IDPAA**F**NDE**F**LADL**F**QHSASND**Y**
TQQATQS**Y**GA**Y**PTQPGQG**Y**SQQSSQP**Y**GQQS**Y**SG**Y**SQSTDTSG**Y**GQSS

NGN2 Wild type

M**F**VKSETLELKEEEDVLVLLGSASPALAALTPLSSSADEEEEEEPGASGGARRQRGAEAGQGA
RGGVAAGAEGCRPARLLGLVHDCKRRPSRARAVSRGAKTAETVQRIKKTRRLKANNRERNRM
HNLNAALDALREVLPT**F**PEDAKLTKIETLR**F**AHN**Y**I**W**ALTETLRLADHCGGGGGGLPGAL**F**SEAV
LLSPGGASAALSSSGDSPSPAST**W**SCTNSPAPSSSVSSNSTSP**Y**SCTLSPASPAGSDMD**YW**Q
PPPPDKHR**Y**APHLPIARDCI

NGN2 AroLITE A

MAVKSETLELKEEEDVLVLLGSASPALAALTPLSSSADEEEEEEPGASGGARRQRGAEAGQGA
RGGVAAGAEGCRPARLLGLVHDCKRRPSRARAVSRGAKTAETVQRIKKTRRLKANNRERNRM
HNLNAALDALREVLPT**F**PEDAKLTKIETLR**F**AHN**Y**I**W**ALTETLRLADHCGGGGGGLPGAL**F**SEAV
LLSPGGASAALSSSGDSPSPASTASCTNSPAPSSSVSSNSTSPASCTLSPASPAGSDMDAAQP
PPPDKHRAAPHLPIARDCI

NGN2 AroPERFECT
M**F**VKSETLELKEEEDVLVLLGSASPALAALTPLSSSADEEEEEEPGASGGARRQRGAEAGQGA
RGGVAAGAEGCRPARLLGLVHDCKRRPSRARAVSRGAKTAETVQRIKKTRRLKANNRERNRM
HNLNAALDALREVLPT**F**PEDAKLTKIETLR**F**AHN**Y**I**W**ALTETLRLADHCGGGGGGLPGAL**F**SEAV
LLSPGGASAA**W**LSSSGDSPSPASTS**Y**CTNSPAPSSSVSSN**Y**STSPSCTLSPASPA**W**GSDMDQ
PPPPDKHR**Y**APHLPIARDCI

NGN2 N-IDR Wild type
M**F**VKSETLELKEEEDVLVLLGSASPALAALTPLSSSADEEEEEEPGASGGARRQRGAEAGQGA
RGGVAAGAEGCRPARLLGLVHDCKRRPSRARAVSRGAKTAETVQRIKKTRRLKANNRERNRM
HNLNAA

NGN2 N-IDR AroPERFECT
M**F**VKSETLELKEEEDV**W**LVLLGSASPALAAL**Y**TPLSSSADEEEEEE**Y**PGASGGARRQRGAE**W**A
GQGARGGVAAGAE**Y**GCRPARLLGLVHDC**W**KRRPSRARAVSRGA**Y**KTAETVQRIKKTRR**Y**LKA
NNRERNRMHNL**W**NAA

NGN2 C-IDR Wild type
AVLLSPGGASAALSSSGDSPSPAST**W**SCTNSPAPSSSVSSNSTSP**Y**SCTLSPASPAGSDMD**YW**
QPPPPDKHR**Y**APHLPIARDCI

NGN2 C-IDR AroLITE A
AVLLSPGGASAALSSSGDSPSPASTASCTNSPAPSSSVSSNSTSPASCTLSPASPAGSDMDAA
QPPPPDKHRAAPHLPIARDCI

NGN2 C-IDR AroPERFECT
AVLLSPGGASAA**W**LSSSGDSPSPASTS**Y**CTNSPAPSSSVSSN**Y**STSPSCTLSPASPA**W**GSDMD
QPPPPDKHR**Y**APHLPIARDCI

MYOD1 Wild type

MELLSPPLRDVDLTAPDGSLCS**F**ATTDD**FY**DDPC**F**DSPDLR**FF**EDLDPRLMHVGALLKPEEHSH
FPAAVHPAPGAREDEHVRAPSGHHQAGRCLL**W**ACKACKRKTTNADRRKAATMRERRRLSKVN
EA**F**ETLKRCTSSNPNQRLPKVEILRNAIR**Y**IEGLQALLRDQDAAPPGAAAA**FY**APGPLPPGRGG
EH**Y**SGDSDASSPRSNCSDGMMD**Y**SGPPSGARRRNC**Y**EGA**YY**NEAPSEPRPGKSAAVSSLDC
LSSIVERISTESPAAPALLLADVPSESPPRRQEAAAPSEGESSGDPTQSPDAAPQCPAGANPNP
I**Y**QVL

MYOD1 AroLITE A

MELLSPPLRDVDLTAPDGSLCSAATTDDAADDPCADSPDLRAAEDLDPRLMHVGALLKPEEHS
HAPAAVHPAPGAREDEHVRAPSGHHQAGRCLLAACKACKRKTTNADRRKAATMRERRRLSKV
NEAAETLKRCTSSNPNQRLPKVEILRNAIRAIEGLQALLRDQDAAPPGAAAAAAAPGPLPPGRG
GEHASGDSDASSPRSNCSDGMMDASGPPSGARRRNCAEGAAANEAPSEPRPGKSAAVSSLD
CLSSIVERISTESPAAPALLLADVPSESPPRRQEAAAPSEGESSGDPTQSPDAAPQCPAGANPN
PIAQVL

MYOD1 AroPERFECT

M**F**ELLSPPLRDVDLTA**F**PDGSLCSATTDDDDP**Y**CDSPDLREDLDPRLM**F**HVGALLKPEEHSHP
A**F**AVHPAPGAREDEHVR**F**APSGHHQAGRCLLAC**F**KACKRKTTNADRRKAATMRERRRLSKVN
EA**F**ETLKRCTSSNPNQRLPKVEILRNAIR**Y**IEGLQALLRDQDAAPPGAAAAAPGPLPPGRGGE**W**
HSGDSDASSPRSNCS**F**DGMMDSGPPSGARRR**Y**NCEGANEAPSEPRPG**Y**KSAAVSSLDCLSS
IV**Y**ERISTESPAAPALLL**Y**ADVPSESPPRRQEAA**Y**APSEGESSGDPTQSP**Y**DAAPQCPAGANPN
PI**Y**QVL

MYOD1 N-IDR Wild type

MELLSPPLRDVDLTAPDGSLCS**F**ATTDD**FY**DDPC**F**DSPDLR**FF**EDLDPRLMHVGALLKPEEHSH
FPAAVHPAPGAREDEHVRAPSGHHQAGRCLL**W**ACKAC

MYOD1 N-IDR AroLITE A

MELLSPPLRDVDLTAPDGSLCSAATTDDAADDPCADSPDLRAAEDLDPRLMHVGALLKPEEHS
HAPAAVHPAPGAREDEHVRAPSGHHQAGRCLLAACKAC

MYOD1 N-IDR AroPERFECT

M**F**ELLSPPLRDVDLTA**F**PDGSLCSATTDDDDP**Y**CDSPDLREDLDPRLM**F**HVGALLKPEEHSHP
A**F**AVHPAPGAREDEHVR**F**APSGHHQAGRCLLAC**F**KAC

MYOD1 C-IDR Wild type
AAAA**FY**APGPLPPGRGGEH**Y**SGDSDASSPRSNCSDGMMD**Y**SGPPSGARRRNC**Y**EGA**YY**NE
APSEPRPGKSAAVSSLDCLSSIVERISTESPAAPALLLADVPSESPPRRQEAAAPSEGESSGDP
TQSPDAAPQCPAGANPNPI**Y**QVL

MYOD1 C-IDR AroLITE A
AAAAAAAPGPLPPGRGGEHASGDSDASSPRSNCSDGMMDASGPPSGARRRNCAEGAAANE
APSEPRPGKSAAVSSLDCLSSIVERISTESPAAPALLLADVPSESPPRRQEAAAPSEGESSGDP
TQSPDAAPQCPAGANPNPIAQVL

MYOD1 C-IDR AroPERFECT
AAAAAPGPLPPGRGGE**W**HSGDSDASSPRSNCS**F**DGMMDSGPPSGARRR**Y**NCEGANEAPSE
PRPG**Y**KSAAVSSLDCLSSIV**Y**ERISTESPAAPALLL**Y**ADVPSESPPRRQEAA**Y**APSEGESSGDP
TQSP**Y**DAAPQCPAGANPNPI**Y**QVL

OCT4 N-IDR Wild type
MAGHLASD**F**A**F**SPPPGGGDGSAGLEPG**W**VDPRT**W**LS**F**QGPPGGPGIGPGSEVLGISPCPPA
YE**F**CGGMA**Y**CGPQVGLGLVPQVGVETLQPEGQAGARVESNSEGTSSEPCADRPNAVKLEKV
EPTPEESQDMKALQKELEQ

OCT4 C-IDR Wild type
KGKRSSIE**W**SQREE**Y**EATGTP**F**PGGAVS**F**PLPPGPH**F**GTPG**Y**GSPH**F**TTL**Y**SVP**F**PEGEA**F**PS
VPVTALGSPMHSN

OCT4 N-IDR AroLITE
MAGHLASDAAASPPPGGGDGSAGLEPGAVDPRTALSAQGPPGGPGIGPGSEVLGISPCPPAA
EACGGMAACGPQVGLGLVPQVGVETLQPEGQAGARVESNSEGTSSEPCADRPNAVKLEKVE
PTPEESQDMKALQKELEQ

OCT4 C-IDR AroLITE

KGKRSSIEASQREEAEATGTPAPGGAVSAPLPPGPHAGTPGAGSPHATTLASVPAPEGEAAPS
VPVTALGSPMHSN

OCT4 N-IDR AroPERFECT
MAGHLASD**F**ASPPPGGGDGSAGL**F**EPGVDPRTLSQGPP**W**GGPGIGPGSEVLGI**W**SPCPPAE
CGGMACG**F**PQVGLGLVPQVGVE**Y**TLQPEGQAGARVES**F**NSEGTSSEPCADRP**Y**NAVKLEKV
EPTPEE**F**SQDMKALQKELEQ

OCT4 C-IDR AroPERFECT
KGKRSSIE**Y**SQREEE**Y**ATGTPP**F**GGAVSP**F**LPPGPH**F**GTPGGS**Y**PHTTLS**F**VPPEGE**Y**APSVP
V**F**TALGSP**F**MHSN

PDX1 IDR Wild type
MNGEEQ**YY**AATQL**Y**KDPCA**F**QRGPAPE**F**SASPPACL**Y**MGRQPPPPPPHP**F**PGALGALEQGS
PPDISP**Y**EVPPLADDPAVAHLHHHLPAQLALPHPPAGP**F**PEGAEPGVLEEPNRVQLP

PDX1 IDR AroLITE
MNGEEQAAAATQLAKDPCAAQRGPAPEASASPPACLAMGRQPPPPPPHPAPGALGALEQGS
PPDISPAEVPPLADDPAVAHLHHHLPAQLALPHPPAGPAPEGAEPGVLEEPNRVQLP

PDX1 IDR AroPERFECT
MN**Y**GEEQAATQLKDPC**Y**AQRGPAPESASPP**Y**ACLMGRQPPPPPP**F**HPPGALGALEQGS**F**PP
DISPEVPPLAD**Y**DPAVAHLHHHLPA**F**QLALPHPPAGPPE**Y**GAEPGVLEEPNRV**F**QLP**F**

FOXA3 IDR Wild type C
RRQKR**F**KLEEKVKKGGSGAATTTRNGTGSAASTTTPAATVTSPPQPPPPAPEPEAQGGEDVG
ALDCGSPASSTP**YF**TGLELPGELKLDAP**Y**N**F**NHP**F**SINNLMSEQTPAPPKLDVG**F**GG**Y**GAEGG
EPGV**YY**QGL**Y**SRSLLNAS

FOXA3 IDR AroLITE C
RRQKRAKLEEKVKKGGSGAATTTRNGTGSAASTTTPAATVTSPPQPPPPAPEPEAQGGEDVG
ALDCGSPASSTPAATGLELPGELKLDAPANANHPASINNLMSEQTPAPPKLDVGAGGAGAEGG
EPGVAAQGLASRSLLNAS

FOXA3 IDR AroPERFECT C

RRQKR**F**KLEEKVKKGGSG**Y**AATTTRNGTGSA**F**ASTTTPAATVTS**Y**PPQPPPPAPEPE**F**AQGG
EDVGALDC**F**GSPASSTPTGLE**F**LPGELKLDAPNN**Y**HPSINNLMSEQT**Y**PAPPKLDVGGGG**Y**AE
GGEPGVQGLS**Y**RSLLNAS

S6Y AroPATCHY1

MSGSSSGSSGGSSSSGSSGSGGSSS**YYYYYYYYYYYYYY**SSGSGSSGSSSGGSSSGSSGSS
SGSSGGSSSSGSSGSGGSSSSSGSGSSGSSSGGSSSGS

S6Y AroPATCHY3

MSGSS**YYYY**SGSSGGSS**YYYY**SSGSSGSGGSSSSSGSGSSGSSSG**YYYY**GSSSGSSGSSS
GSSGGSSSSGSSGSGGSSSSSGSGSSGSSSGGSSSGSSS

S6Y AroPERFECT

MSGSSSG**Y**SSGGSS**Y**SSGSSG**Y**SGGSSS**Y**SSGSGS**Y**SGSSSG**Y**GSSSGS**Y**SGSSSG**Y**SSG
GSS**Y**SSGSSG**Y**SGGSSS**Y**SSGSGS**Y**SGSSSG**Y**GSSSGS**Y**

D6Y AroPERFECT

MSDDDSG**Y**SSDGDS**Y**SDDSDG**Y**SDDSDS**Y**DSDSDS**Y**DGSDDG**Y**GDDSDS**Y**DGDDSG**Y**SDG
DDS**Y**SSDDDG**Y**SDGDSD**Y**SDDDGS**Y**DGDSDG**Y**GSDDGD**Y**

For Extended Data Figure 2i, the following sequences were used:

HOXD4 IDR Wild type

MVMSS**Y**MVNSK**Y**VDPK**F**PPCEE**Y**LQGG**Y**LGEQGAD**YY**GGGAQGAD**F**QPPGL**Y**PRPD**F**GEQ
P**F**GGSGPGPGSALPARGHGQEPGGPGGH**Y**AAPGEPCPAPPAPPPAPLPGARA**Y**SQSDPKQP
PSGTALKQPAVV**Y**P**W**MKKVVSRGP**Y**SIVSPKC

HOXD4 IDR AroLITE A

MVMSSAMVNSKAVDPKAPPCEEALQGGALGEQGADAAGGGAQGADAQPPGLAPRPDAGEQ
PAGGSGPGPGSALPARGHGQEPGGPGGHAAAPGEPCPAPPAPPPAPLPGARAASQSDPKQP
PSGTALKQPAVVAPAMKKVVSRGP**Y**SIVSPKC

HOXD4 IDR AroLITE S

MVMSSSMVNSKSVDPKSPPCEESLQGGSLGEQGADSSGGGAQGADSQPPGLSPRPDSGEQ
PSGGSGPGPGSALPARGHGQEPGGPGGHSAAPGEPCPAPPAPPPAPLPGARASSQSDPKQP
PSGTALKQPAVVSPSMKKVVSRGP**Y**SIVSPKC

HOXD4 IDR AroLITE G

MVMSSGMVNSKGVDPKGPPCEEGLQGGGLGEQGADGGGGGAQGADGQPPGLGPRPDGG
EQPGGGSGPGPGSALPARGHGQEPGGPGGHGAAPGEPCPAPPAPPPAPLPGARAGSQSDP
KQPPSGTALKQPAVVGPGMKKVVSRGP**Y**SIVSPKC

Supplementary Figures

Supplementary Figure 1

a

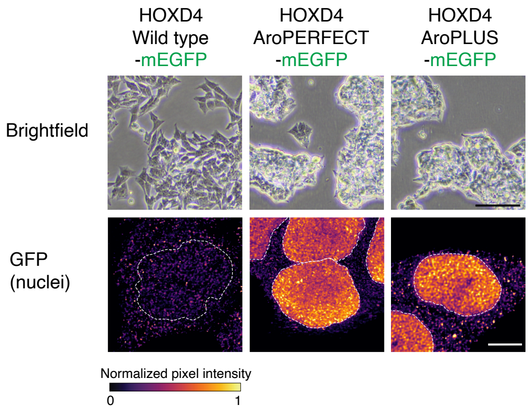

HAP1 knock-in cells, Independent clones of the same genotypes as cell lines shown in Figure 3a.

b

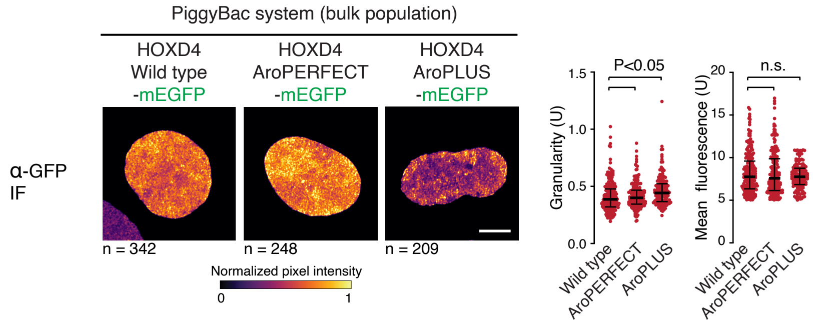

HAP1PiggyBac cell population Independently generated from the cell population shown in Figure 3b-c (same population as shwon below in panel c)

c

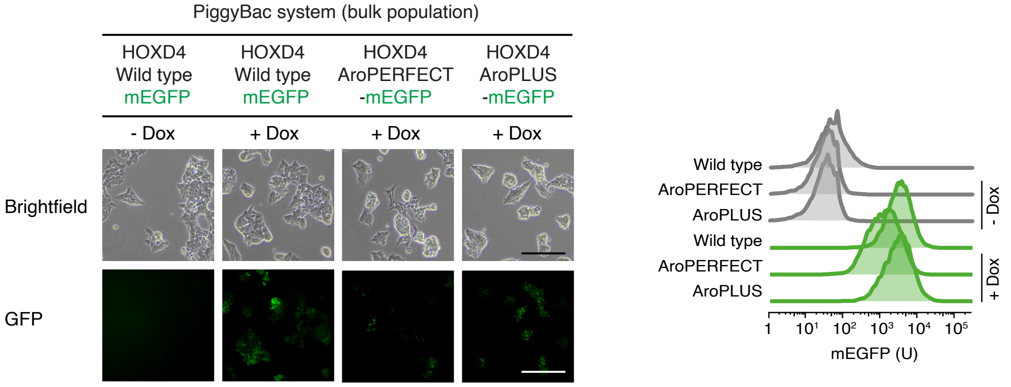

HAP1PiggyBac cell population Independently generated from the cell population shown in Extended Data Figure 6g.

Supplementary Figure 1. Phenotypes of HOXD4-modified cells in independent cultures.

a. (top) Differential interference contrast microscopy of the indicated cell lines. Scale bar is 0.4 mm. (bottom) Representative fluorescence microscopy images of cell nuclei. The fusion proteins were visualized using anti-GFP immunofluorescence in fixed cells. The normalized signal intensity was calculated by dividing standard deviation of mEGFP signal of each nucleus by the corresponding mean mEGFP signal.

b. (left) Representative images of HAP1 HOXD4 wild type-mEGFP, HOXD4 AroPERFECT-mEGFP and HOXD4 AroPLUS-mEGFP nuclei after 24h of HOXD4 expression. The fusion proteins were visualized using mEGFP fluorescence in fixed cells. The normalized signal intensity was calculated by dividing standard deviation of mEGFP signal of each nucleus by the corresponding mean mEGFP signal. Number of individual nuclei per condition is displayed. (right) Granularity scores of nuclei, with corresponding mean nuclear mEGFP intensities. Data are displayed as mean ± SD for individual nuclei from two biological replicates. *P*-values are two-sided unpaired t-tests.

c. (top) Differential interference contrast microscopy of the indicated cell lines. Scale bar is 0.4 mm. (bottom) Fluorescence microscopy images. Cells were imaged 14 days after constant doxycycline induction. Scale bar is 10μm. (right) Flow cytometry analysis of mEGFP expression in HAP1 HOXD4-mEGFP PiggyBac bulk cell populations after 14 days of Dox induction. A representative quantification is shown. Data normalized to mode.

Supplementary Figure 2

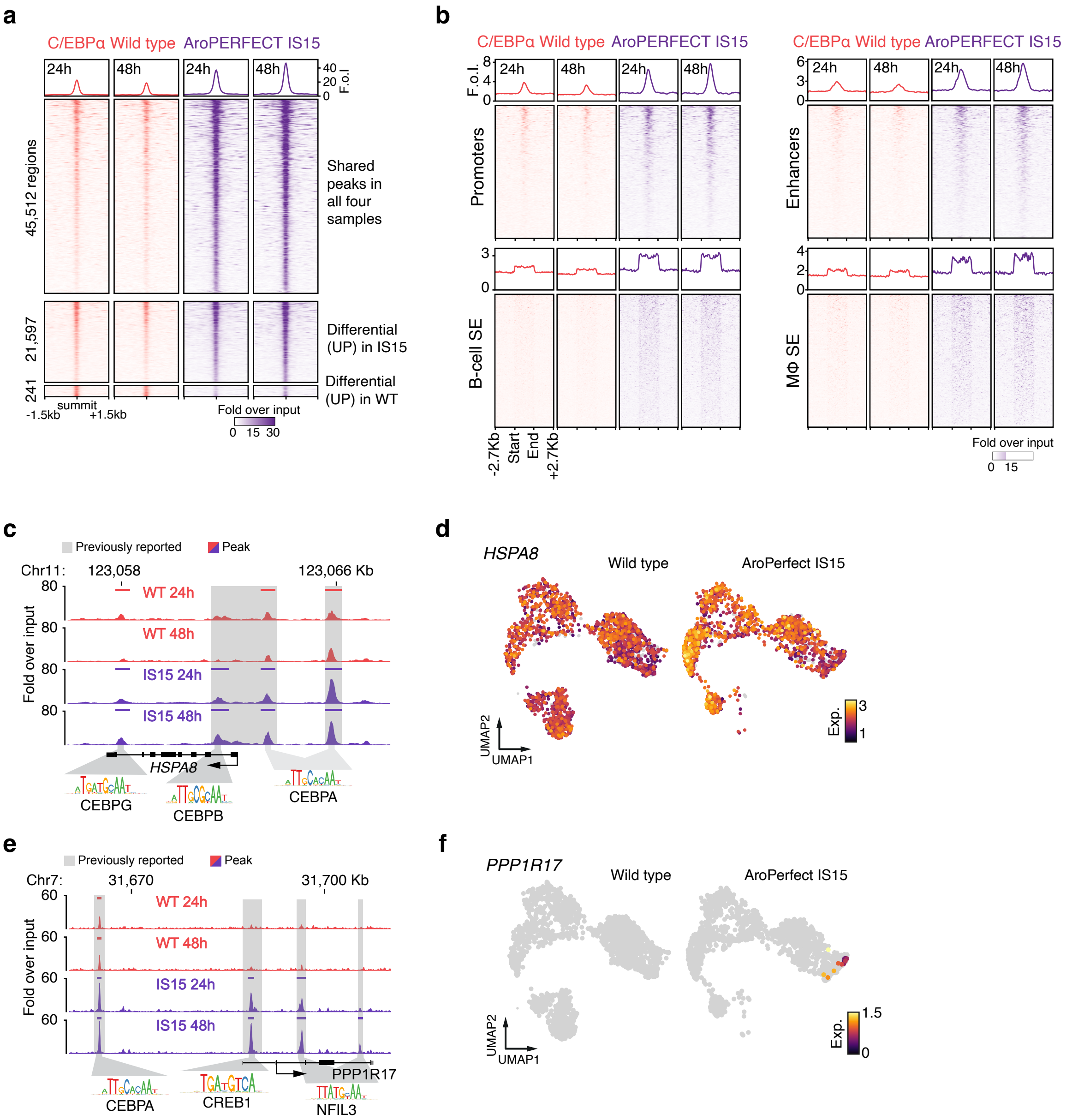

Supplementary Figure 2. C/EBPα ChIP-seq supporting data.

a. Heatmap representation of ChIP-Seq read densities of C/EBPα Wild type and AroPERFECT IS15 within a 1.5kb window around all shared C/EBPα peaks (top), differentially enriched peaks in C/EBPα AroPERFECT IS15 (center) and differentially enriched peaks in C/EBPα Wild type (bottom). F.o.I: fold over input.

b. Heatmap representation of ChIP-Seq read densities of C/EBPα wild type and AroPERFECT IS15 at 24h and 48h after induction of C/EBPα overexpression. Signal is shown within a 2.7kb window around gene promoters (top left), enhancers (top right), B-cell super-enhancers (bottom left) and macrophage super-enhancers (bottom right). Regions between the start and end co-ordinates were length-normalized. F.o.I: fold over input.

c,e. C/EBPα AroPERFECT IS15 shows enhanced binding at the *HSPA8* (**e**) and *PPP1R17* (**g**) loci. Displayed are genome browser tracks of ChIP-Seq data, 24 and 48 hours after C/EBPα induction. Co-ordinates are hg38 genome assembly co-ordinates. F.o.I: fold over input.

d,f. UMAPs colored on *HSPA8* (**f**) and *PPP1R17* (**h**) expression.

Supplementary Figure 3

a

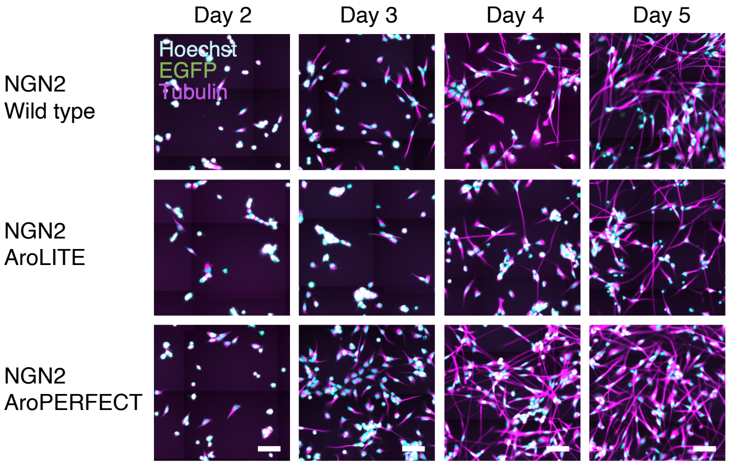

Independent clone set #2
The experiments were performed on independently generated clones of the same genotypes as the cell used in Figure 6d-f

b

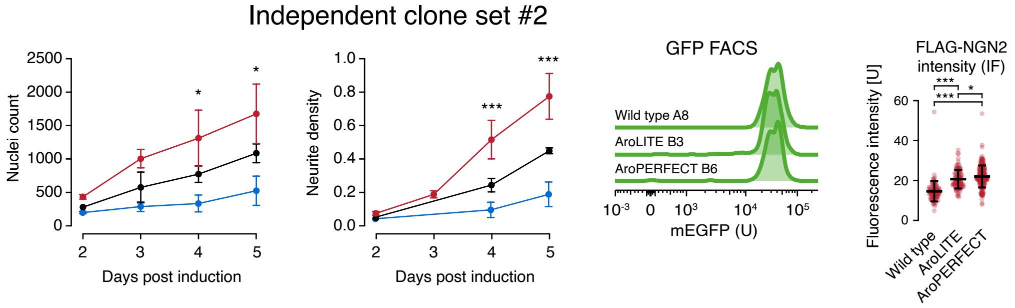

Independent clone set #2
The experiments were performed on independently generated clones of the same genotypes as the cell used in Figure 6d-f

c

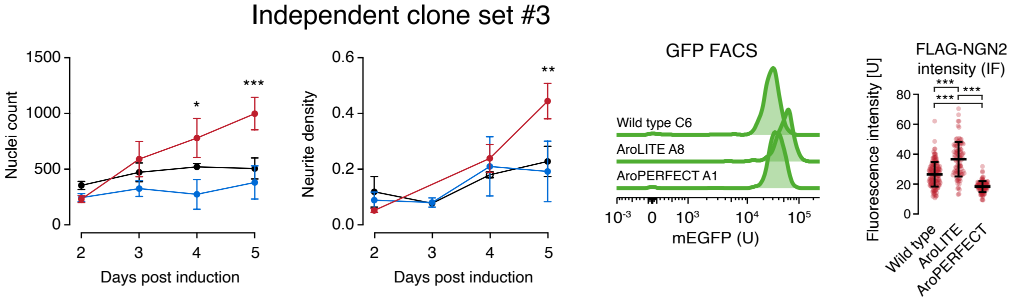

Independent clone set #3
The experiments were performed on independently generated clones of the same genotypes as the cell used in Figure 6d-f

Supplementary Figure 3. Phenotypes of NGN2-modified cells in independent cultures.

a. Representative fluorescence microscopy images of differentiating human iPSCs expressing the indicated NGN2 proteins. Tubulin staining is in magenta, nuclear counterstain (Hoechst) in blue, NGN2-T2A-mEGFP is green. Scale bar is 0.1 mm.

b-c. Quantifications of experiments performed of independent clone series #2 (**b**), and #3 (**c**).

(far left) Quantification of the number of cells based on Hoechst nuclear staining in the NGN2-directed differentiation experiments. Data are displayed as mean ± SD.

(left) Quantification of neurite density based on tubulin staining in the NGN2-directed differentiation experiments. Data are displayed as mean ± SD.

(right) Flow cytometry analysis of mEGFP expression. Data normalized to mode.

(far right) Quantification of FLAG-NGN2 signal in fixed cell immunofluorescence images.

P-values are from one-way multi-comparison ANOVA.

P-values in panel b: nuclei count: $P_{(\text{Wild type day4 vs. AroPERFECT day4})}$=0.0292 , $P_{(\text{Wild type day5 vs. AroPERFECT day5})}$= 0.0218, neurite density: $P_{(\text{Wild type day4 vs. AroPERFECT day4})}$<0.0001 , $P_{(\text{Wild type day5 vs. AroPERFECT day5})}$<0.0001. Fluorescence intensity: $P_{(\text{Wild type vs. AroLITE})}$<0.0001, $P_{(\text{Wild type vs. AroPERFECT})}$<0.0001, $P_{(\text{AroLITE vs. AroPERFECT})}$=0.0338.

P-values in panel c:: nuclei count: $P_{(\text{Wild type day4 vs. AroPERFECT day4})}$=0.01130 , $P_{(\text{Wild type day5 vs. AroPERFECT day5})}$= 0.00022, neurite density: $P_{(\text{Wild type day4 vs. AroPERFECT day4})}$=0.9104 , $P_{(\text{Wild type day5 vs. AroPERFECT day5})}$=0.0016. Fluorescence intensity: $P_{(\text{Wild type vs. AroLITE})}$<0.0001, $P_{(\text{Wild type vs. AroPERFECT})}$<0.0001, $P_{(\text{AroLITE vs. AroPERFECT})}$<0.0001.

Supplementary Figure 4

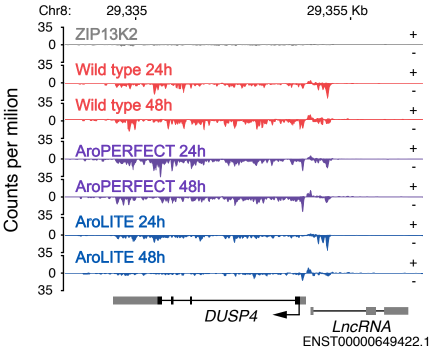

TT-SLAM-Seq data at additional example loci in the NGN2-induced neural differentiation system. *SOX2, POU5F1* and *NANOG* are examples for embryonic genes.

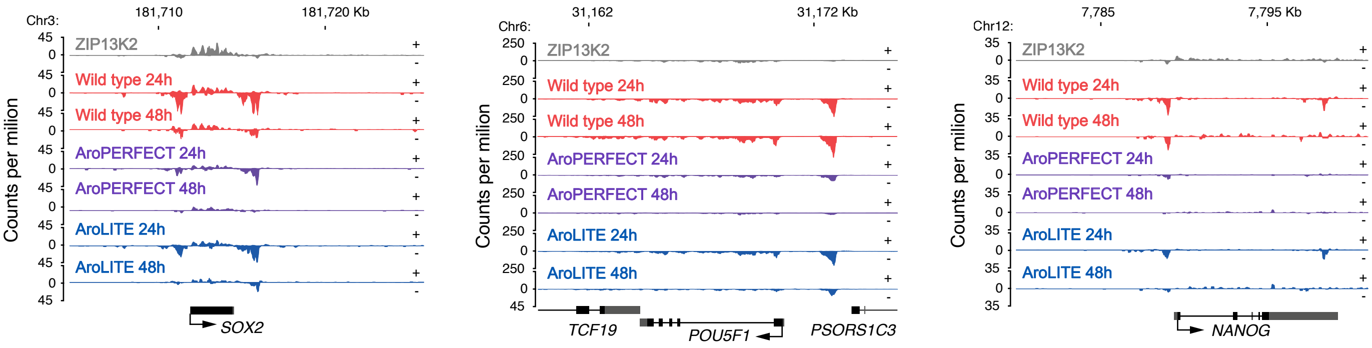

Supplementary Figure 4. TT-SLAM-Seq data at additional example loci.

TT-SLAM-Seq data at the indicated loci. See also Extended Data Fig. 9k-l.
