# Supplementary material for: An activity-specificity trade-off encoded in human transcription factors
Source: Nat Cell Biol. 2024 Jul 5;26(8):1309–21. doi: 10.1038/s41556-024-01411-0 (PMC11321997; doi:10.1038/s41556-024-01411-0)
Supplement: Supplementary file 2 — Reporting Summary [file 41556_2024_1411_MOESM2_ESM.pdf]

nature portfolio

Corresponding author(s): Denes Hnisz

Last updated by author(s): Feb 5, 2024

Reporting Summary

Nature Portfolio wishes to improve the reproducibility of the work that we publish. This form provides structure for consistency and transparency in reporting. For further information on Nature Portfolio policies, see our Editorial Policies and the Editorial Policy Checklist.

Statistics

For all statistical analyses, confirm that the following items are present in the figure legend, table legend, main text, or Methods section.

n/a	Confirmed	
☐	☒	The exact sample size (*n*) for each experimental group/condition, given as a discrete number and unit of measurement
☒	☐	A statement on whether measurements were taken from distinct samples or whether the same sample was measured repeatedly
☐	☒	The statistical test(s) used AND whether they are one- or two-sided<br>*Only common tests should be described solely by name; describe more complex techniques in the Methods section.*
☒	☐	A description of all covariates tested
☐	☒	A description of any assumptions or corrections, such as tests of normality and adjustment for multiple comparisons
☐	☒	A full description of the statistical parameters including central tendency (e.g. means) or other basic estimates (e.g. regression coefficient) AND variation (e.g. standard deviation) or associated estimates of uncertainty (e.g. confidence intervals)
☐	☒	For null hypothesis testing, the test statistic (e.g. *F*, *t*, *r*) with confidence intervals, effect sizes, degrees of freedom and *P* value noted<br>*Give P values as exact values whenever suitable.*
☒	☐	For Bayesian analysis, information on the choice of priors and Markov chain Monte Carlo settings
☒	☐	For hierarchical and complex designs, identification of the appropriate level for tests and full reporting of outcomes
☒	☐	Estimates of effect sizes (e.g. Cohen's *d*, Pearson's *r*), indicating how they were calculated

Our web collection on statistics for biologists contains articles on many of the points above.

Software and code

Policy information about availability of computer code

Data collection

- Fluorescence images were collected with widefield and confocal microscopes using Zen Black 2.3. software (Zeiss).

- Western blot images were collected using Image Lab software (version 6.1.0 buildt 7) (Bio-Rad).

- FACS data was collected with FACS Diva software (BD Biosciences) and BD Aria II and BD Celesta instruments.

Data analysis

Fluorescence images were analyzed using ZenBlue 3.1, 3.2, 3.4 (Zeiss), Fiji/ImageJ (2.1.0/1.53i). Data was plotted using GraphPad PRISM 9.

FACS data was analyzed using FlowJo (v10.7)

GraphPad PRISM (v9.2.0) was used for statistical analysis and barplot generation.

For single cell RNA-seq analysis, the following software were used: Cell ranger v3.1.0, Seurat 4.0.6, R v4.2., velocyto v1.0, scvelo v0.3.1, DESeq2 v1.41.0.

For Bulk RNA-seq data analysis, the following software were used: cutadapt 4.7, STAR aligner 2.7.9a, DESeq2 v1.41.0, R v4.2, GSEAPreranked v6.0.12.

For Chip-seq cutadapt 4.7, bwa v0.7.17, samtools v1.19.1, gatk v4.4, MACs v3.0b1, bamCoverage v3.5.1, MEME v5.1.1, deepTools2 v3.5.1, bamCoverage v3.5.1, deepTools2 v3.5.1, Bigwigmerge v377, bedGraphToBigWig v377, pygenometracks 3.7, DiffBind v3.6.5 bedtools v2.6.0.

For TT-SLAM-seq analysis, the following software were used: STAR aligner 2.7.9a, seqtk 1.3-r106, SLAM-DUNK v0.4.1, samtools v1.19.1, featureCounts v2.0.6, bamCoverage v3.5.1, deepTools2 v3.5.1, Bigwigmerge v377, bedGraphToBigWig v377, pygenometracks 3.7.

Protein sequence analysis was performed with the following software: Biostrings v2.40.2, Metapredict v2, UCSF ChimeraX v1.6, Alphafold v2.0, MEME v5.1.1, PLAAC v1, localCIDER 0.1.20,

All software versions and parameters are listed in the Methods.

Custom Python and R code is available https://github.com/hniszlab/TFsubopt.

The Periodic Block finder code is made available in the link https://github.com/alexpmagalhaes/PeriodicBlock_finder.

The QuasiIDRfinder code is made available in the link https://github.com/gozdekibar/QuasiIDRFinder

Custom code available under : https://doi.org/10.5281/zenodo.10628753

For manuscripts utilizing custom algorithms or software that are central to the research but not yet described in published literature, software must be made available to editors and reviewers. We strongly encourage code deposition in a community repository (e.g. GitHub). See the Nature Portfolio guidelines for submitting code & software for further information.

Data

Policy information about availability of data

All manuscripts must include a data availability statement. This statement should provide the following information, where applicable:

- Accession codes, unique identifiers, or web links for publicly available datasets
- A description of any restrictions on data availability
- For clinical datasets or third party data, please ensure that the statement adheres to our policy

Sequencing data was deposited at the Gene Expression Omnibus (GEO), under the accession ID: GSE201655.

RAW data was deposited at: https://owww.molgen.mpg.de/~TFsuboptimization/

For NGS experiments of human samples we used human genome hg38 and annotations from GENCODE GRCh38.p13.

For NGS experiments of mouse samples we used mouse mm10 genome and annotations from GENCODE GRCm38.p6.

For annotation of proteins and IDRs we used GENCODE gene annotation v39, GENCODE GRCh38.p13 and IDs from Ensembl v104.

Transcription factor sequences and annotations were from AnimalTFDB3.0.

Field-specific reporting

Please select the one below that is the best fit for your research. If you are not sure, read the appropriate sections before making your selection.

☒ Life sciences ☐ Behavioural & social sciences ☐ Ecological, evolutionary & environmental sciences

For a reference copy of the document with all sections, see nature.com/documents/nr-reporting-summary-flat.pdf

Life sciences study design

All studies must disclose on these points even when the disclosure is negative.

Sample size

No statistical methods were used to predetermine sample sizes. Sample sizes are indicated in the figure panels or legends or in the Methods. For droplet experiments we imaged at least 10 independent fields of a view for each experimental condition based on current methodology in the field (Sabari et al. Science. 2018; Boija et al. Cell. 2018). For imaging experiments multiple replicate experiments were performed indiciated in the legends, according to current standards in the field (Sabari et al., Science 2018; Boija et al. Cell 2018). For transactivation experiments, we collected luminescence data from at least four independent transfections of two biological replicates for each experimental condition. For fluorescence imaging of differentiating neurons and muscle cells, we imaged 3-5 randomly selected fields of view per biological replicate and experimental condition in three independent rounds of differentiation. For Supplemental Figure 3 we imaged two clonal lines with 3-5 randomly selected fields of view and displayed them separately to highlight possible clonal heterogeneity.

Data exclusions

In rare instances, out of focus images or images with the majority of cells washed off the slide by mechanical force (e.g. pipetting) were excluded in differentiation experiments.

Replication

For droplet experiments we imaged at least 3-5 fields of view in at least 2 independent replicate series.
For transactivation experiments, we collected luminescence data from at least four independent transfections of two biological replicates for each experimental condition.
For fluorescence imaging of differentiating neurons and muscle cells, we imaged 3-5 randomly selected fields of view per biological replicate and experimental condition in three independent rounds of differentiation. For Supplemental Data 3 we imaged two clonal lines with 3-5 randomly selected fields of view and displayed them separately to highlight possible clonal heterogeneity. For this experiment, replication attempts were successful for 3/5 cases, two attempts failed on technical reasons. For all experiments attempts at replication were generally successful, unless technical issues arose.
The number of replicates are reported in figures and legends.

Randomization | Not relevant for the study.

Blinding | Blinding was not relevant for the experiments.

Reporting for specific materials, systems and methods

We require information from authors about some types of materials, experimental systems and methods used in many studies. Here, indicate whether each material, system or method listed is relevant to your study. If you are not sure if a list item applies to your research, read the appropriate section before selecting a response.

Materials & experimental systems

n/a	Involved in the study
☐	☒ Antibodies
☐	☒ Eukaryotic cell lines
☒	☐ Palaeontology and archaeology
☒	☐ Animals and other organisms
☒	☐ Human research participants
☒	☐ Clinical data
☒	☐ Dual use research of concern

Methods

n/a	Involved in the study
☐	☒ ChIP-seq
☐	☒ Flow cytometry
☒	☐ MRI-based neuroimaging

Antibodies

Antibodies used

Immunofluorescence experiments:

GFP (A11122, 1:500, Invitrogen)
FLAG (F1804, 1:500, Sigma-Aldrich)
Donkey anti-Rabbit-Alexa647 (711-605-152, 1:1000,JacksonImmuno)
Donkey anti-Mouse-Alexa647 (715-605-150, 1:1000,JacksonImmuno)

Western blotting experiments:

HSP90 (BD610419; 1:4000, BD)
IFI16 (sc-8023, 1:200, Santa Cruz)
GFP (A11122, 1:2000, Invitrogen)
ARHGAP4 (sc-376251, 1:200, Santa Cruz)
ESX1 (sc-365740, 1:200, Santa Cruz)
FLAG (F1804, 1:2000, Sigma-Aldrich)
GATA6 (AF1700, 1:1000, RnD)
Peroxidase-AffiniPure Donkey Anti-Goat IgG (705-035-147, 1:5000, JacksonImmuno)
Peroxidase IgG Fraction Monoclonal Mouse Anti-Rabbit IgG (211-032-171, 1:5000, JacksonImmuno)
Peroxidase AffiniPure Goat Anti-Mouse IgG (115-035-174, 1:1000, JacksonImmuno)
Gal4 (sc-510, 1:200, Santa Cruz)

FACS experiments:

CD19 APC-Cy7 Mouse anti-Human CD19 (557791, 1:200, BD)
APC Mouse Anti-Human CD11b/Mac-1 (550019, 1:200, BD)
CD66a Alexa Fluor 647 anti-human CD66a (398905, 1:250, BioLegend)
FCGR2A PE anti-human FCGR2A (305503, 1:200, BioLegend)

ChIP-seq experiments:

GFP clone 3E6 (A-11120, 1:500, Invitrogen)
FLAG (F1804, 1:250, Sigma-Aldrich)

Validation

Antibodies in Immunofluorescence, ChIP-seq and Western blot experiments were validated by comparing to parental cell lines without transgene expression.

All antibodies are validated by the provider and cited in numerous publications:

Immunofluorescence and Western blot experiments:

GFP (A11122, Invitrogen) – rabbit
https://www.thermofisher.com/antibody/product/A-11122.html?CID=AFLCA-A-11122

FLAG (F1804, Sigma-Aldrich) – mouse
https://www.sigmaaldrich.com/deepweb/assets/sigmaaldrich/product/documents/119/160/f1804bul-mk.pdf

HSP90 (BD610419, BD) – mouse

https://wwwfishersci.com/shop/products/anti-hsp90-clone-68-bd-2/BDB610419

IFI16 (sc-8023, Santa Cruz) – mouse
https://datasheets.scbt.com/sc-8023.pdf

ARHGAP4 (sc-376251, Santa Cruz) – mouse
https://datasheets.scbt.com/sc-376251.pdf

ESX1 (sc-365740, Santa Cruz) – mouse
https://datasheets.scbt.com/sc-365740.pdf

GATA6 (AF1700, RnD) – mouse
https://resources.rndsystems.com/pdfs/datasheets/af1700.pdf?v=20240206

Gal4 (sc-510, Santa Cruz) – mouse
https://datasheets.scbt.com/sc-510.pdf

FACS experiments:

CD19 APC-Cy7 Mouse anti-Human CD19 (557791, BD)
https://www.bdbiosciences.com/content/bdb/paths/generate-tds-document.us.557791.pdf

APC Mouse Anti-Human CD11b/Mac-1 (550019, BD)
https://www.bdbiosciences.com/content/bdb/paths/generate-tds-document.de.550019.pdf

CD66a Alexa Fluor 647 anti-human CD66a (398905, BioLegend)
https://www.biolegend.com/en-us/products/alexa-fluor-647-anti-human-cd66a-b-c-e-antibody-20073

FCGR2A PE anti-human FCGR2A (305503, BioLegend)
https://www.biolegend.com/de-at/products/pe-anti-human-fcgr2a-cd32a-antibody-21510?GroupID=GROUP28

ChiP-seq experiments:

GFP clone 3E6 (A-11120, Invitrogen)
https://www.thermofisher.com/antibody/product/GFP-Antibody-clone-3E6-Monoclonal/A-11120

Eukaryotic cell lines

Policy information about cell lines

Cell line source(s)

General information provided in methods under: "Cell culture"

- V6.5 mouse embryonic stem cells (mESCs), source: Konrad Hochedlinger lab

- HEK293T, source: ATCC, Identifier: CRL-3216

-SH-SY5Y, source: DSMZ, Identifier: ACC-209

-Kelly, source: DSMZ, Identifier: ACC-355

-HAP1, source: Aktas Lab (MPI-MG)

-HAP1-HOXD4-mEGFP lines, source: This paper (see Methods "Generation of HOXD4 GFP knock-in and knockout lines" and "Generation of Doxycycline-inducible HOXD4 overexpression lines in HAP1 cells")

-ZIP13K2, source: Müller Lab (MPI-MG)

-ZiP13K2-NGN2-T2A-mEGFP lines, source: This paper (see Methods "Generation of Doxycycline-inducible NGN2 overexpression systems in human iPS cells")

-C2C12, source: Stricker Lab (Freie Universität Berlin)

-C2C12-MYOD1-T2A-mEGFP lines, source: This paper (see Methods "Generation of Doxycycline-inducible MYOD1 overexpression lines in C2C12 cells")

-RCH-rtTA, source: Graf Lab (CRG Barcelona)

-RCH-rtTA-CEBPa-GFP lines, source: This paper (see Methods "Generation of Doxycycline-inducible C/EBPα overexpression lines in RCH cells")

-U2OS, source: Kinkley Lab (MPI-MG)

Authentication

The identity of HEK293T, SH-SY5Y, Kelly, HAP1, RCH-rtTA, U2Os, C2C12,parental V6.5 mESCs and ZIP13K2 iPSCs, and all cell

Authentication	lines derived from them has been validated using<br>morphological characteristics, qPCRs, FACS, immunofluorescence, RNA-seq, and marker gene expression (where applicable) but have not been authenticated.<br>Expression of HOXD4, NGN2, MYOD1, CEBPa transgene expression was validated by FACS and RNA-sequencing.
Mycoplasma contamination	All cell lines tested negative for mycoplasma contamination.
Commonly misidentified lines<br>(See ICLAC register)	None used.

ChIP-seq

Data deposition

☒ Confirm that both raw and final processed data have been deposited in a public database such as GEO.

☐ Confirm that you have deposited or provided access to graph files (e.g. BED files) for the called peaks.

Data access links
May remain private before publication.

Sequencing data was deposited at the Gene Expression Omnibus (GEO), under the accession ID: GSE201655

Files in database submission

Processed Files

GSM6069079_DH-RNA-043barcodes.tsv.gz
GSM6069079_DH-RNA-043features.tsv.gz
GSM6069079_DH-RNA-043matrix.mtx.gz
GSM6069080_DH-RNA-044barcodes.tsv.gz
GSM6069080_DH-RNA-044features.tsv.gz
GSM6069080_DH-RNA-044matrix.mtx.gz
GSM6069081_DH-RNA-045barcodes.tsv.gz
GSM6069081_DH-RNA-045features.tsv.gz
GSM6069081_DH-RNA-045matrix.mtx.gz
GSM6710791_DH-RNA-065_hg38.star.ReadsPerGene.out.tab.gz
GSM6710792_DH-RNA-066_hg38.star.ReadsPerGene.out.tab.gz
GSM6710793_DH-RNA-067_hg38.star.ReadsPerGene.out.tab.gz
GSM6710794_DH-RNA-068_hg38.star.ReadsPerGene.out.tab.gz
GSM6710795_DH-RNA-069_hg38.star.ReadsPerGene.out.tab.gz
GSM6710796_DH-RNA-070_hg38.star.ReadsPerGene.out.tab.gz
GSM6710797_DH-RNA-071_hg38.star.ReadsPerGene.out.tab.gz
GSM6710798_DH-RNA-072_hg38.star.ReadsPerGene.out.tab.gz
GSM6710799_DH-RNA-073_hg38.star.ReadsPerGene.out.tab.gz
GSM6710800_DH-RNA-074_hg38.star.ReadsPerGene.out.tab.gz
GSM6710801_DH-RNA-075_hg38.star.ReadsPerGene.out.tab.gz
GSM6710802_DH-RNA-076_hg38.star.ReadsPerGene.out.tab.gz
GSM6710803_DH-RNA-077_hg38.star.ReadsPerGene.out.tab.gz
GSM6710804_DH-RNA-078_hg38.star.ReadsPerGene.out.tab.gz
GSM6710805_DH-RNA-079_hg38.star.ReadsPerGene.out.tab.gz
GSM6710806_ZIP13K2_WT_1_HG38_NGN2.star.ReadsPerGene.out.tab.gz
GSM6710807_ZIP13K2_WT_2_HG38_NGN2.star.ReadsPerGene.out.tab.gz
GSM6710808_ZIP13K2_WT_3_HG38_NGN2.star.ReadsPerGene.out.tab.gz
GSM6710809_NGN2_WT_1_HG38_NGN2.star.ReadsPerGene.out.tab.gz
GSM6710810_NGN2_WT_2_HG38_NGN2.star.ReadsPerGene.out.tab.gz
GSM6710811_NGN2_WT_3_HG38_NGN2.star.ReadsPerGene.out.tab.gz
GSM6710812_NGN2_AroLITE_1_HG38_NGN2.star.ReadsPerGene.out.tab.gz
GSM6710813_NGN2_AroLITE_2_HG38_NGN2.star.ReadsPerGene.out.tab.gz
GSM6710814_NGN2_AroLITE_3_HG38_NGN2.star.ReadsPerGene.out.tab.gz
GSM6710815_NGN2_AroPERFECT_1_HG38_NGN2.star.ReadsPerGene.out.tab.gz
GSM6710816_NGN2_AroPERFECT_2_HG38_NGN2.star.ReadsPerGene.out.tab.gz
GSM6710817_NGN2_AroPERFECT_3_HG38_NGN2.star.ReadsPerGene.out.tab.gz
GSM6710818_C2C12_WT_1_mm39_myod1.star.ReadsPerGene.out.tab.gz
GSM6710819_C2C12_WT_2_mm39_myod1.star.ReadsPerGene.out.tab.gz
GSM6710820_C2C12_WT_3_mm39_myod1.star.ReadsPerGene.out.tab.gz
GSM6710821_MYOD1_AroLite_1_mm39_myod1.star.ReadsPerGene.out.tab.gz
GSM6710822_MYOD1_AroLite_2_mm39_myod1.star.ReadsPerGene.out.tab.gz
GSM6710823_MYOD1_AroLite_3_mm39_myod1.star.ReadsPerGene.out.tab.gz
GSM6710824_MYOD1_AroLiteC_1_mm39_myod1.star.ReadsPerGene.out.tab.gz
GSM6710825_MYOD1_AroLiteC_2_mm39_myod1.star.ReadsPerGene.out.tab.gz
GSM6710826_MYOD1_AroLiteC_3_mm39_myod1.star.ReadsPerGene.out.tab.gz
GSM6710827_MYOD1_AroPerfect_1_mm39_myod1.star.ReadsPerGene.out.tab.gz
GSM6710828_MYOD1_AroPerfect_2_mm39_myod1.star.ReadsPerGene.out.tab.gz
GSM6710829_MYOD1_AroPerfect_3_mm39_myod1.star.ReadsPerGene.out.tab.gz
GSM6710830_MYOD1_AroPerfectC_1_mm39_myod1.star.ReadsPerGene.out.tab.gz
GSM6710831_MYOD1_AroPerfectC_2_mm39_myod1.star.ReadsPerGene.out.tab.gz
GSM6710832_MYOD1_AroPerfectC_3_mm39_myod1.star.ReadsPerGene.out.tab.gz

GSM6710833_MYOD1_WT_1_mm39_myod1.star.ReadsPerGene.out.tab.gz
GSM6710834_MYOD1_WT_2_mm39_myod1.star.ReadsPerGene.out.tab.gz
GSM6710835_MYOD1_WT_3_mm39_myod1.star.ReadsPerGene.out.tab.gz
GSM6710836_WT_24_ChIP_rep1_peaks.narrowPeak.gz
GSM6710837_WT_24_ChIP_rep2_peaks.narrowPeak.gz
GSM6710838_WT_48_ChIP_rep1_peaks.narrowPeak.gz
GSM6710839_WT_48_ChIP_rep2_peaks.narrowPeak.gz
GSM6710840_IS15_24_ChIP_rep1_peaks.narrowPeak.gz
GSM6710841_IS15_24_ChIP_rep2_peaks.narrowPeak.gz
GSM6710842_IS15_48_ChIP_rep1_peaks.narrowPeak.gz
GSM6710843_IS15_48_ChIP_rep2_peaks.narrowPeak.gz
GSM6710852_ZIP13K2_NGN2_24h_AroLITE_2_peaks.narrowPeak.gz
GSM6710853_ZIP13K2_NGN2_24h_AroLITE_3_peaks.narrowPeak.gz
GSM6710854_ZIP13K2_NGN2_24h_AroPERFECT_2_peaks.narrowPeak.gz
GSM6710855_ZIP13K2_NGN2_24h_AroPERFECT_3_peaks.narrowPeak.gz
GSM6710856_ZIP13K2_NGN2_24h_WT_1_peaks.narrowPeak.gz
GSM6710857_ZIP13K2_NGN2_24h_WT_2_peaks.narrowPeak.gz
GSM6710858_ZIP13K2_NGN2_48h_AroLITE_1_peaks.narrowPeak.gz
GSM6710859_ZIP13K2_NGN2_48h_AroLITE_3_peaks.narrowPeak.gz
GSM6710860_ZIP13K2_NGN2_48h_AroLITE_2_peaks.narrowPeak.gz
GSM6710861_ZIP13K2_NGN2_48h_AroPERFECT_1_peaks.narrowPeak.gz
GSM6710862_ZIP13K2_NGN2_48h_AroPERFECT_3_peaks.narrowPeak.gz
GSM6710863_ZIP13K2_NGN2_48h_AroPERFECT_2_peaks.narrowPeak.gz
GSM6710864_ZIP13K2_NGN2_48h_WT_1_peaks.narrowPeak.gz
GSM6710865_ZIP13K2_NGN2_48h_WT_2_peaks.narrowPeak.gz
GSM6710866_ZIP13K2_NGN2_48h_WT_3_peaks.narrowPeak.gz

RAW Files

mpimg_L23394-1_DH-RNA-043_S1_L001_R1_001.fastq.gz
mpimg_L23394-1_DH-RNA-043_S1_L002_I1_001.fastq.gz
mpimg_L23395-1_DH-RNA-044_S2_L002_I1_001.fastq.gz
mpimg_L23394-1_DH-RNA-043_S1_L002_R1_001.fastq.gz
mpimg_L23396-1_DH-RNA-045_S3_L001_R1_001.fastq.gz
mpimg_L23394-1_DH-RNA-043_S1_L002_R2_001.fastq.gz
mpimg_L23396-1_DH-RNA-045_S3_L001_I1_001.fastq.gz
mpimg_L23395-1_DH-RNA-044_S2_L001_R1_001.fastq.gz
mpimg_L23396-1_DH-RNA-045_S3_L002_I1_001.fastq.gz
mpimg_L23394-1_DH-RNA-043_S1_L001_R2_001.fastq.gz
mpimg_L23396-1_DH-RNA-045_S3_L001_R2_001.fastq.gz
mpimg_L23395-1_DH-RNA-044_S2_L001_I1_001.fastq.gz
mpimg_L23395-1_DH-RNA-044_S2_L001_R2_001.fastq.gz
mpimg_L23394-1_DH-RNA-043_S1_L001_I1_001.fastq.gz
mpimg_L23395-1_DH-RNA-044_S2_L002_R1_001.fastq.gz
mpimg_L23395-1_DH-RNA-044_S2_L002_R2_001.fastq.gz
mpimg_L23396-1_DH-RNA-045_S3_L002_R1_001.fastq.gz
mpimg_L23396-1_DH-RNA-045_S3_L002_R2_001.fastq.gz

mpimg_L24588-1_DH-RNA-065_S65_R1_001.fastq.gz
mpimg_L24589-1_DH-RNA-066_S66_R1_001.fastq.gz
mpimg_L24590-1_DH-RNA-067_S67_R1_001.fastq.gz
mpimg_L24591-1_DH-RNA-068_S68_R1_001.fastq.gz
mpimg_L24592-1_DH-RNA-069_S69_R1_001.fastq.gz
mpimg_L24593-1_DH-RNA-070_S70_R1_001.fastq.gz
mpimg_L24594-1_DH-RNA-071_S71_R1_001.fastq.gz
mpimg_L24595-1_DH-RNA-072_S72_R1_001.fastq.gz
mpimg_L24596-1_DH-RNA-073_S73_R1_001.fastq.gz
mpimg_L24597-1_DH-RNA-074_S74_R1_001.fastq.gz
mpimg_L24598-1_DH-RNA-075_S75_R1_001.fastq.gz
mpimg_L24599-1_DH-RNA-076_S76_R1_001.fastq.gz
mpimg_L24600-1_DH-RNA-077_S77_R1_001.fastq.gz
mpimg_L24601-1_DH-RNA-078_S78_R1_001.fastq.gz
mpimg_L24602-1_DH-RNA-079_S79_R1_001.fastq.gz
mpimg_L24588-1_DH-RNA-065_S65_R2_001.fastq.gz
mpimg_L24589-1_DH-RNA-066_S66_R2_001.fastq.gz
mpimg_L24590-1_DH-RNA-067_S67_R2_001.fastq.gz
mpimg_L24591-1_DH-RNA-068_S68_R2_001.fastq.gz
mpimg_L24592-1_DH-RNA-069_S69_R2_001.fastq.gz
mpimg_L24593-1_DH-RNA-070_S70_R2_001.fastq.gz
mpimg_L24594-1_DH-RNA-071_S71_R2_001.fastq.gz
mpimg_L24595-1_DH-RNA-072_S72_R2_001.fastq.gz
mpimg_L24596-1_DH-RNA-073_S73_R2_001.fastq.gz
mpimg_L24597-1_DH-RNA-074_S74_R2_001.fastq.gz
mpimg_L24598-1_DH-RNA-075_S75_R2_001.fastq.gz
mpimg_L24599-1_DH-RNA-076_S76_R2_001.fastq.gz
mpimg_L24600-1_DH-RNA-077_S77_R2_001.fastq.gz

mpimg_L24601-1_DH-RNA-078_S78_R2_001.fastq.gz
mpimg_L24602-1_DH-RNA-079_S79_R2_001.fastq.gz

mpimg_L26725-1_DH-RNA-093_S416_R1_001.fastq.gz
mpimg_L26726-1_DH-RNA-094_S417_R1_001.fastq.gz
mpimg_L26727-1_DH-RNA-095_S418_R1_001.fastq.gz
mpimg_L26728-1_DH-RNA-096_S419_R1_001.fastq.gz
mpimg_L26729-1_DH-RNA-097_S420_R1_001.fastq.gz
mpimg_L26730-1_DH-RNA-098_S421_R1_001.fastq.gz
mpimg_L26731-1_DH-RNA-099_S422_R1_001.fastq.gz
mpimg_L26732-1_DH-RNA-100_S423_R1_001.fastq.gz
mpimg_L26733-1_DH-RNA-101_S424_R1_001.fastq.gz
mpimg_L26734-1_DH-RNA-102_S425_R1_001.fastq.gz
mpimg_L26735-1_DH-RNA-103_S426_R1_001.fastq.gz
mpimg_L26736-1_DH-RNA-104_S427_R1_001.fastq.gz
mpimg_L26725-1_DH-RNA-093_S416_R2_001.fastq.gz
mpimg_L26726-1_DH-RNA-094_S417_R2_001.fastq.gz
mpimg_L26727-1_DH-RNA-095_S418_R2_001.fastq.gz
mpimg_L26728-1_DH-RNA-096_S419_R2_001.fastq.gz
mpimg_L26729-1_DH-RNA-097_S420_R2_001.fastq.gz
mpimg_L26730-1_DH-RNA-098_S421_R2_001.fastq.gz
mpimg_L26731-1_DH-RNA-099_S422_R2_001.fastq.gz
mpimg_L26732-1_DH-RNA-100_S423_R2_001.fastq.gz
mpimg_L26733-1_DH-RNA-101_S424_R2_001.fastq.gz
mpimg_L26734-1_DH-RNA-102_S425_R2_001.fastq.gz
mpimg_L26735-1_DH-RNA-103_S426_R2_001.fastq.gz
mpimg_L26736-1_DH-RNA-104_S427_R2_001.fastq.gz

mpimg_L26564-1_DH-RNA-063_S22_R1_001.fastq.gz
mpimg_L26565-1_DH-RNA-064_S23_R1_001.fastq.gz
mpimg_L26566-1_DH-RNA-080_S24_R1_001.fastq.gz
mpimg_L26567-1_DH-RNA-081_S25_R1_001.fastq.gz
mpimg_L26568-1_DH-RNA-082_S26_R1_001.fastq.gz
mpimg_L26569-1_DH-RNA-083_S27_R1_001.fastq.gz
mpimg_L26570-1_DH-RNA-084_S28_R1_001.fastq.gz
mpimg_L26571-1_DH-RNA-085_S44_R1_001.fastq.gz
mpimg_L26572-1_DH-RNA-XXX_S46_R1_001.fastq.gz
mpimg_L27772-1_DH-RNA-108_S4_R1_001.fastq.gz
mpimg_L27773-1_DH-RNA-109_S5_R1_001.fastq.gz
mpimg_L27774-1_DH-RNA-110_S6_R1_001.fastq.gz
mpimg_L26573-1_DH-RNA-087_S47_R1_001.fastq.gz
mpimg_L26574-1_DH-RNA-088_S48_R1_001.fastq.gz
mpimg_L26575-1_DH-RNA-089_S49_R1_001.fastq.gz
mpimg_L26576-1_DH-RNA-090_S50_R1_001.fastq.gz
mpimg_L26577-1_DH-RNA-091_S51_R1_001.fastq.gz
mpimg_L26578-1_DH-RNA-092_S52_R1_001.fastq.gz
mpimg_L26564-1_DH-RNA-063_S22_R2_001.fastq.gz
mpimg_L26565-1_DH-RNA-064_S23_R2_001.fastq.gz
mpimg_L26566-1_DH-RNA-080_S24_R2_001.fastq.gz
mpimg_L26567-1_DH-RNA-081_S25_R2_001.fastq.gz
mpimg_L26568-1_DH-RNA-082_S26_R2_001.fastq.gz
mpimg_L26569-1_DH-RNA-083_S27_R2_001.fastq.gz
mpimg_L26570-1_DH-RNA-084_S28_R2_001.fastq.gz
mpimg_L26571-1_DH-RNA-085_S44_R2_001.fastq.gz
mpimg_L26572-1_DH-RNA-XXX_S46_R2_001.fastq.gz
mpimg_L27772-1_DH-RNA-108_S4_R2_001.fastq.gz
mpimg_L27773-1_DH-RNA-109_S5_R2_001.fastq.gz
mpimg_L27774-1_DH-RNA-110_S6_R2_001.fastq.gz
mpimg_L26573-1_DH-RNA-087_S47_R2_001.fastq.gz
mpimg_L26574-1_DH-RNA-088_S48_R2_001.fastq.gz
mpimg_L26575-1_DH-RNA-089_S49_R2_001.fastq.gz
mpimg_L26576-1_DH-RNA-090_S50_R2_001.fastq.gz
mpimg_L26577-1_DH-RNA-091_S51_R2_001.fastq.gz
mpimg_L26578-1_DH-RNA-092_S52_R2_001.fastq.gz

D6105_lib_07222AAD_ACTTCGTT-GATGCGTT_R1_001.fastq.gz
W24C_2_10106AAD_CTGCCAAG-TCCATATA_R1_001.fastq.gz
D6107_lib_07224AAD_ATGAGAGG-TCGTCTTG_R1_001.fastq.gz
W48C_2_10108AAD_CGCCAGTC-CCAAGACG_R1_001.fastq.gz
D6106_lib_07223AAD_CGGTTGGT-TGCAGCGT_R1_001.fastq.gz
P24C_2_10107AAD_ACGCCGCA-ATGTTAAC_R1_001.fastq.gz
D6108_lib_07225AAD_CTCGCAAG-GATCTACG_R1_001.fastq.gz
P48C_2_10109AAD_CTAAACAA-TCGCTACG_R1_001.fastq.gz
D6109_lib_07226AAD_GATCTTGC-CCAATTCC_R1_001.fastq.gz
W24I_2_10110AAD_TATACCTC-TGTGACTA_R1_001.fastq.gz
D6111_lib_07228AAD_TAACGCCA-AGGCAAGA_R1_001.fastq.gz

W48I_2_10112AAD_ACTCTTAG-AATCCACG_R1_001.fastq.gz
D6110_lib_07227AAD_TCAGATAC-CGCGAGAC_R1_001.fastq.gz
P24I_2_10111AAD_CTCTTGAT-CCACTTCT_R1_001.fastq.gz
D6112_lib_07229AAD_GTCAACCA-ATATGCAA_R1_001.fastq.gz
P48I_2_10113AAD_GAGCAACA-GCATCTAC_R1_001.fastq.gz

mpimg_L27298-1_DH-Other-170_S158_R1_001.fastq.gz
mpimg_L27299-1_DH-Other-171_S255_R1_001.fastq.gz
mpimg_L27301-1_DH-Other-173_S257_R1_001.fastq.gz
mpimg_L27302-1_DH-Other-174_S258_R1_001.fastq.gz
mpimg_L27296-1_DH-Other-168_S156_R1_001.fastq.gz
mpimg_L27297-1_DH-Other-169_S157_R1_001.fastq.gz
mpimg_L27290-1_DH-Other-162_S150_R1_001.fastq.gz
mpimg_L27291-1_DH-Other-163_S151_R1_001.fastq.gz
mpimg_L27292-1_DH-Other-164_S152_R1_001.fastq.gz
mpimg_L27293-1_DH-Other-165_S153_R1_001.fastq.gz
mpimg_L27294-1_DH-Other-166_S154_R1_001.fastq.gz
mpimg_L27295-1_DH-Other-167_S155_R1_001.fastq.gz
mpimg_L27287-1_DH-Other-159_S147_R1_001.fastq.gz
mpimg_L27288-1_DH-Other-160_S148_R1_001.fastq.gz
mpimg_L27289-1_DH-Other-161_S149_R1_001.fastq.gz
mpimg_L27305-1_DH-Other-177_S261_R1_001.fastq.gz
mpimg_L27306-1_DH-Other-178_S262_R1_001.fastq.gz
mpimg_L27304-1_DH-Other-176_S260_R1_001.fastq.gz
mpimg_L27298-1_DH-Other-170_S158_R2_001.fastq.gz
mpimg_L27299-1_DH-Other-171_S255_R2_001.fastq.gz
mpimg_L27301-1_DH-Other-173_S257_R2_001.fastq.gz
mpimg_L27302-1_DH-Other-174_S258_R2_001.fastq.gz
mpimg_L27296-1_DH-Other-168_S156_R2_001.fastq.gz
mpimg_L27297-1_DH-Other-169_S157_R2_001.fastq.gz
mpimg_L27290-1_DH-Other-162_S150_R2_001.fastq.gz
mpimg_L27291-1_DH-Other-163_S151_R2_001.fastq.gz
mpimg_L27292-1_DH-Other-164_S152_R2_001.fastq.gz
mpimg_L27293-1_DH-Other-165_S153_R2_001.fastq.gz
mpimg_L27294-1_DH-Other-166_S154_R2_001.fastq.gz
mpimg_L27295-1_DH-Other-167_S155_R2_001.fastq.gz
mpimg_L27287-1_DH-Other-159_S147_R2_001.fastq.gz
mpimg_L27288-1_DH-Other-160_S148_R2_001.fastq.gz
mpimg_L27289-1_DH-Other-161_S149_R2_001.fastq.gz
mpimg_L27305-1_DH-Other-177_S261_R2_001.fastq.gz
mpimg_L27306-1_DH-Other-178_S262_R2_001.fastq.gz
mpimg_L27304-1_DH-Other-176_S260_R2_001.fastq.gz

Genome browser session
(e.g. UCSC)

Reviewers can view the ChIP-Seq data at:
https://genome-euro.ucsc.edu/s/apmagalhaes/SubOpt_ChIP

Methodology

Replicates

ChIP-Seq experiments were performed with 3 replicates for FLAG-NGN2 and CEBP/a. Bulk RNA-seq and TTSLAMseq experiments were performed with 3 biological replicates.

Sequencing depth

Total reads Uniquely mapped reads Length of reads Type Library
83642894 54949626 100bp Pair-end HAP1_Parental_WT_rep1
71027423 47610277 100bp Pair-end HAP1_Parental_WT_rep2
56951635 38986064 100bp Pair-end HAP1_Parental_WT_rep3
56955224 39329753 100bp Pair-end HAP1_HOXD4_WT_GFP_rep1
62124406 41271109 100bp Pair-end HAP1_HOXD4_WT_GFP_rep2
65732072 44132421 100bp Pair-end HAP1_HOXD4_WT_GFP_rep3
72989824 49331533 100bp Pair-end HAP1_HOXD4_AroPERFECT_GFP_rep1
75091733 48798291 100bp Pair-end HAP1_HOXD4_AroPERFECT_GFP_rep2
64293354 43258227 100bp Pair-end HAP1_HOXD4_AroPERFECT_GFP_rep3
63446798 43938397 100bp Pair-end HAP1_HOXD4_AroPLUS_GFP_rep1
84882022 53853668 100bp Pair-end HAP1_HOXD4_AroPLUS_GFP_rep2
67722437 40444092 100bp Pair-end HAP1_HOXD4_AroPLUS_GFP_rep3
104794729 53935807 100bp Pair-end HAP1_HOXD4_KO_rep1
72708520 42280338 100bp Pair-end HAP1_HOXD4_KO_rep2
85083118 37090405 100bp Pair-end HAP1_HOXD4_KO_rep3

78214706 61657120 100bp Pair-end ZIP13K2_WT_1
71899108 56656736 100bp Pair-end ZIP13K2_WT_2
59160286 46943262 100bp Pair-end ZIP13K2_WT_3
84741214 59010761 100bp Pair-end NGN2_WT_1
85396044 58477095 100bp Pair-end NGN2_WT_2
84726168 60119354 100bp Pair-end NGN2_WT_3

72071817 58259440 100bp Pair-end NGN2_AroLITE_1
67907275 53515875 100bp Pair-end NGN2_AroLITE_2
67771801 55279375 100bp Pair-end NGN2_AroLITE_3
88930215 70855293 100bp Pair-end NGN2_AroPERFECT_1
93952187 75443215 100bp Pair-end NGN2_AroPERFECT_2
84778537 68363391 100bp Pair-end NGN2_AroPERFECT_3

42321075 29857940 100bp Pair-end C2C12_WT_1
64598444 46267749 100bp Pair-end C2C12_WT_2
82306985 59149573 100bp Pair-end C2C12_WT_3
80946669 63282013 100bp Pair-end MYOD1_AroLite_1
74033673 56197975 100bp Pair-end MYOD1_AroLite_2
58269310 44063566 100bp Pair-end MYOD1_AroLite_3
46052693 36169911 100bp Pair-end MYOD1_AroLiteC_1
49238600 37820987 100bp Pair-end MYOD1_AroLiteC_2
33461352 25298292 100bp Pair-end MYOD1_AroLiteC_3
59090530 55449892 100bp Pair-end MYOD1_AroPerfect_1
59553741 57216936 100bp Pair-end MYOD1_AroPerfect_2
67867623 64948740 100bp Pair-end MYOD1_AroPerfect_3
30402772 20460464 100bp Pair-end MYOD1_AroPerfectC_1
32400056 21473188 100bp Pair-end MYOD1_AroPerfectC_2
37670706 26130992 100bp Pair-end MYOD1_AroPerfectC_3
30345574 21932837 100bp Pair-end MYOD1_WT_1
32627684 24050399 100bp Pair-end MYOD1_WT_2
23852795 16502519 100bp Pair-end MYOD1_WT_3

45083745 44443621 50 bp Single end WT_24_ChIP_rep1
47757874 47104678 50 bp Single end WT_24_ChIP_rep2
46055986 45487287 50 bp Single end WT_48_ChIP_rep1
45561595 45349108 50 bp Single end WT_48_ChIP_rep2
45467504 45044928 50 bp Single end IS15_24_ChIP_rep1
47081785 46445180 50 bp Single end IS15_24_ChIP_rep2
50391760 49809562 50 bp Single end IS15_48_ChIP_rep1
47302784 46575423 50 bp Single end IS15_48_ChIP_rep2
45972769 45491878 50 bp Single end WT_24_ChIP_input_rep1
48567463 48270072 50 bp Single end WT_24_ChIP_input_rep2
46889096 46423177 50 bp Single end WT_48_ChIP_input_rep1
48057746 47646638 50 bp Single end WT_48_ChIP_input_rep2
46780833 46170463 50 bp Single end IS15_24_ChIP_input_rep1
49335393 49039314 50 bp Single end IS15_24_ChIP_input_rep2
45899181 45056772 50 bp Single end IS15_48_ChIP_input_rep1
46974517 46887338 50 bp Single end IS15_48_ChIP_input_rep2

51712478 51678156 100bp Pair-end AroLITE_24_2
61068607 61002674 100bp Pair-end AroLITE_24_3
74767419 74688631 100bp Pair-end AroPERFECT_24_2
66344050 66286721 100bp Pair-end AroPERFECT_24_3
65551957 65496639 100bp Pair-end NGN2_24_1
69964691 69909977 100bp Pair-end NGN2_24_2
45990628 45958665 100bp Pair-end AroLITE_48_1
52756925 52715022 100bp Pair-end AroLITE_48_2
56012770 55971775 100bp Pair-end AroLITE_48_3
47400492 47361689 100bp Pair-end AroPERFECT_48_1
40986292 40953444 100bp Pair-end AroPERFECT_48_2
48033066 47995766 100bp Pair-end AroPERFECT_48_3
59224278 59179536 100bp Pair-end NGN2_48_1
51833944 51790572 100bp Pair-end NGN2_48_2
55064698 55025027 100bp Pair-end NGN2_48_3
60332885 60260788 100bp Pair-end NGN2_AroLITE_input
52295769 52226140 100bp Pair-end NGN2_AroPERFECT_input
72991162 72903923 100bp Pair-end NGN2_WT_input

22813448 2066101 100bp Single-end ZIP13K2_r1
23694568 2680532 100bp Single-end ZIP13K2_r2
95313445 15277183 100bp Single-end NGN2_WT_12h_r1
63274105 9573508 100bp Single-end NGN2_WT_12h_r2
63464230 9373107 100bp Single-end NGN2_WT_12h_r3
84398829 14917632 100bp Single-end NGN2_WT_24h_r1
76779317 15055873 100bp Single-end NGN2_WT_24h_r2
87319181 16552717 100bp Single-end NGN2_WT_24h_r3
77479680 10457921 100bp Single-end NGN2_AroPERFECT_12h_r1
63477115 9794869 100bp Single-end NGN2_AroPERFECT_12h_r2
75570606 11082568 100bp Single-end NGN2_AroPERFECT_12h_r3
72830923 13159432 100bp Single-end NGN2_AroPERFECT_24h_r2
95041489 16849381 100bp Single-end NGN2_AroPERFECT_24h_r3
89364578 12395003 100bp Single-end NGN2_AroLITE_12h_r1

75578998 11261735 100bp Single-end NGN2_AroLITE_12h_r2
89349138 12255078 100bp Single-end NGN2_AroLITE_12h_r3
83998939 14418922 100bp Single-end NGN2_AroLITE_24h_r2
66904097 12572224 100bp Single-end NGN2_AroLITE_24h_r3

Antibodies

For FLAG-NGN2 ChIP-seq in ZIP13K2 cells FLAG (F1804, 1:2000). For C/EBPa ChIP-seq in RCH-rtTA cells GFP clone 3E6 (A-11120).

Peak calling parameters

Raw reads of treatment and input samples were subjected to adapter and quality trimming with cutadapt (version 2.4; parameters: --nextseq-trim 20 --overlap 5 --minimum-length 25 --adapter AGATCGGAAGAGC -A AGATCGGAAGAGC). Reads were aligned separately to the mouse genome (mm10) or human genome (hg38) using bwa with the 'mem' command (version v0.7.17, default parameters). A sorted BAM file was obtained and indexed using samtools with the 'sort' and 'index' commands (version 1.10). Duplicate reads were identified and removed using gatk (version 4.1.4.1) with the 'MarkDuplicates' command and default parameters. Technical replicates of treatment and input samples were merged respectively using samtools 'merge'.
Peaks were called with reads aligning to the mouse genome only using MACS3 'callpeak' (version 3.0.8 b1; parameters --bdg --SPMR) using the input samples as control samples.
Genome-wide coverage tracks for single and merged replicates normalized by library size and input signal was subtracted using MACS3 output.

Data quality

Quality of raw reads was assessed using FastQC. Reads were trimmed using cutadapt in order to remove low-quality bases and adapter content.

% total deduplicated percentage

62.83 WT_24_ChIP_rep1
73.07 WT_24_ChIP_rep2
74.94 WT_48_ChIP_rep1
81.54 WT_48_ChIP_rep2
75.95 IS15_24_ChIP_rep1
76.73 IS15_24_ChIP_rep2
72.00 IS15_48_ChIP_rep1
72.56 IS15_48_ChIP_rep2

80.22 WT_24_ChIP_rep1_Input
64.69 WT_24_ChIP_rep2_Input
77.28 WT_48_ChIP_rep1_Input
63.18 WT_48_ChIP_rep2_Input
79.23 IS15_24_ChIP_rep1_Input
63.96 IS15_24_ChIP_rep2_Input
78.34 IS15_48_ChIP_rep1_Input
66.27 IS15_48_ChIP_rep2_Input

66.29 ZIP13K2_NGN2_24h_AroLITE_2_Input
67.99 ZIP13K2_NGN2_24h_AroLITE_2
66.29 ZIP13K2_NGN2_24h_AroLITE_3_Input
67.02 ZIP13K2_NGN2_24h_AroLITE_3
62.07 ZIP13K2_NGN2_24h_AroPERFECT_1_Input
58.45 ZIP13K2_NGN2_24h_AroPERFECT_1
62.07 ZIP13K2_NGN2_24h_AroPERFECT_2_Input
67.34 ZIP13K2_NGN2_24h_AroPERFECT_2
62.07 ZIP13K2_NGN2_24h_AroPERFECT_3_Input
69.38 ZIP13K2_NGN2_24h_AroPERFECT_3
55.76 ZIP13K2_NGN2_24h_WT_1_Input
72.08 ZIP13K2_NGN2_24h_WT_1
55.76 ZIP13K2_NGN2_24h_WT_2_Input
63.96 ZIP13K2_NGN2_24h_WT_2
66.29 ZIP13K2_NGN2_48h_AroLITE_1_Input
61.48 ZIP13K2_NGN2_48h_AroLITE_1
66.29 ZIP13K2_NGN2_48h_AroLITE_2_Input
53.67 ZIP13K2_NGN2_48h_AroLITE_2
66.29 ZIP13K2_NGN2_48h_AroLITE_3_Input
60.54 ZIP13K2_NGN2_48h_AroLITE_3
62.07 ZIP13K2_NGN2_48h_AroPERFECT_1_Input
67.40 ZIP13K2_NGN2_48h_AroPERFECT_1
62.07 ZIP13K2_NGN2_48h_AroPERFECT_2_Input
69.76 ZIP13K2_NGN2_48h_AroPERFECT_2
62.07 ZIP13K2_NGN2_48h_AroPERFECT_3_Input
65.80 ZIP13K2_NGN2_48h_AroPERFECT_3
55.76 ZIP13K2_NGN2_48h_WT_1_Input
72.81 ZIP13K2_NGN2_48h_WT_1
55.76 ZIP13K2_NGN2_48h_WT_2_Input
73.15 ZIP13K2_NGN2_48h_WT_2
55.76 ZIP13K2_NGN2_48h_WT_3_Input
71.64 ZIP13K2_NGN2_48h_WT_3
61.25 ZIP13K2_WT_1_Input
40.92 ZIP13K2_WT_1

61.25 ZIP13K2_WT_2_Input
27.87 ZIP13K2_WT_2
61.25 ZIP13K2_WT_3_Input
18.67 ZIP13K2_WT_3

Software

cutadapt
bwa mem
Star Aligner
samtools
gatk
MACS3
bamCoverage
SLAM-DUNK
featureCounts
seqtk

Flow Cytometry

Plots

Confirm that:

☒ The axis labels state the marker and fluorochrome used (e.g. CD4-FITC).

☒ The axis scales are clearly visible. Include numbers along axes only for bottom left plot of group (a 'group' is an analysis of identical markers).

☒ All plots are contour plots with outliers or pseudocolor plots.

☐ A numerical value for number of cells or percentage (with statistics) is provided.

Methodology

Sample preparation

Cells were fixed for 15 minutes in 4% PFA at room temperature. This was followed by two washes in PBS. Flow cytometry workflow for RCH-rtTA cells provided in methods under "C/EBPα mediated B-cell to macrophage transdifferentiation" and "FACS analysis of CD66a and FCGR2A during C/EBPα-mediated B-cell to macrophage differentiation"

Instrument

BD FACS Celesta

Software

FACS Diva for collection and FlowJo for analysis

Cell population abundance

Cell population abundance is represented as normalized mode

Gating strategy

Gating for negative and positive population was determined with untreated or isotype controls.

☒ Tick this box to confirm that a figure exemplifying the gating strategy is provided in the Supplementary Information.
