# Supplementary figures and images for: An activity-specificity trade-off encoded in human transcription factors
Source: Nat Cell Biol. 2024 Jul 5;26(8):1309–21. doi: 10.1038/s41556-024-01411-0 (PMC11321997; doi:10.1038/s41556-024-01411-0)

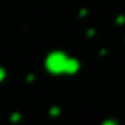

Supplement: Supplementary file 5 — Fusion event between mEGFP-tagged HOXD4 wild-type IDR droplets in vitro. [file 41556_2024_1411_MOESM5_ESM.gif]

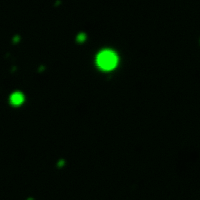

Supplement: Supplementary file 6 — mEGFP-tagged HOXD4 wild-type IDR in vitro droplets wetting the surface of the microscopy slide. [file 41556_2024_1411_MOESM6_ESM.gif]

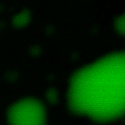

Supplement: Supplementary file 7 — Fusion event between mEGFP-tagged HOXB1 wild-type IDR droplets in vitro. [file 41556_2024_1411_MOESM7_ESM.gif]

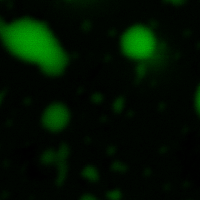

Supplement: Supplementary file 8 — mEGFP-tagged HOXB1 wild-type IDR in vitro droplets wetting the surface of the microscopy slide. [file 41556_2024_1411_MOESM8_ESM.gif]

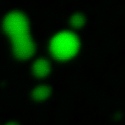

Supplement: Supplementary file 9 — Fusion event between mEGFP-tagged HOXC4 wild-type IDR droplets in vitro. [file 41556_2024_1411_MOESM9_ESM.gif]

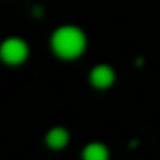

Supplement: Supplementary file 10 — mEGFP-tagged HOXC4 wild-type IDR in vitro droplets wetting the surface of the microscopy slide. [file 41556_2024_1411_MOESM10_ESM.gif]

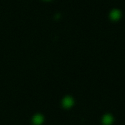

Supplement: Supplementary file 11 — Fusion event between mEGFP-tagged HOXD4 AroPERFECT IDR droplets in vitro. [file 41556_2024_1411_MOESM11_ESM.gif]

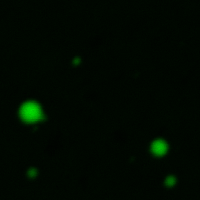

Supplement: Supplementary file 12 — mEGFP-tagged HOXD4 AroPERFECT IDR in vitro droplets wetting the surface of the microscopy slide. [file 41556_2024_1411_MOESM12_ESM.gif]

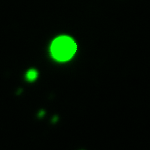

Supplement: Supplementary file 13 — Fusion event between mEGFP-tagged C/EBPα wild-type IDR droplets in vitro. [file 41556_2024_1411_MOESM13_ESM.gif]

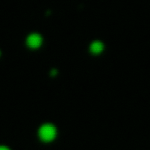

Supplement: Supplementary file 14 — Fusion event between mEGFP-tagged C/EBPα AroPERFECT IS15 IDR droplets in vitro. [file 41556_2024_1411_MOESM14_ESM.gif]

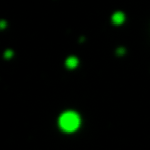

Supplement: Supplementary file 15 — Fusion event between mEGFP-tagged C/EBPα AroPERFECT IS10 IDR droplets in vitro. [file 41556_2024_1411_MOESM15_ESM.gif]

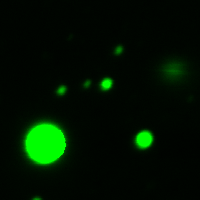

Supplement: Supplementary file 16 — mEGFP-tagged C/EBPα wild-type IDR in vitro droplets wetting the surface of the microscopy slide. [file 41556_2024_1411_MOESM16_ESM.gif]

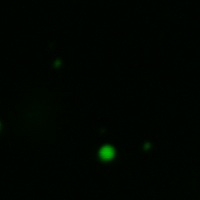

Supplement: Supplementary file 17 — mEGFP-tagged C/EBPα AroPERFECT IS15 IDR in vitro droplets wetting the surface of the microscopy slide. [file 41556_2024_1411_MOESM17_ESM.gif]

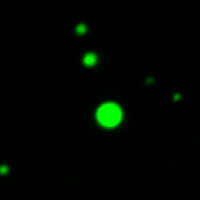

Supplement: Supplementary file 18 — mEGFP-tagged C/EBPα AroPERFECT IS10 IDR in vitro droplets wetting the surface of the microscopy slide. [file 41556_2024_1411_MOESM18_ESM.gif]

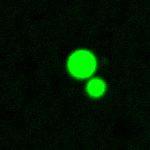

Supplement: Supplementary file 19 — Fusion event between mEGFP-tagged NGN2 wild-type IDR droplets in vitro. [file 41556_2024_1411_MOESM19_ESM.gif]

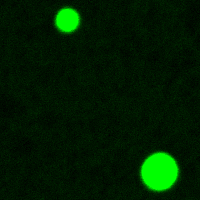

Supplement: Supplementary file 20 — mEGFP-tagged NGN2 wild-type IDR in vitro droplets wetting the surface of the microscopy slide. [file 41556_2024_1411_MOESM20_ESM.gif]

Source Data Figure 3

Uncropped blot images for Figure 3f

Rep 1

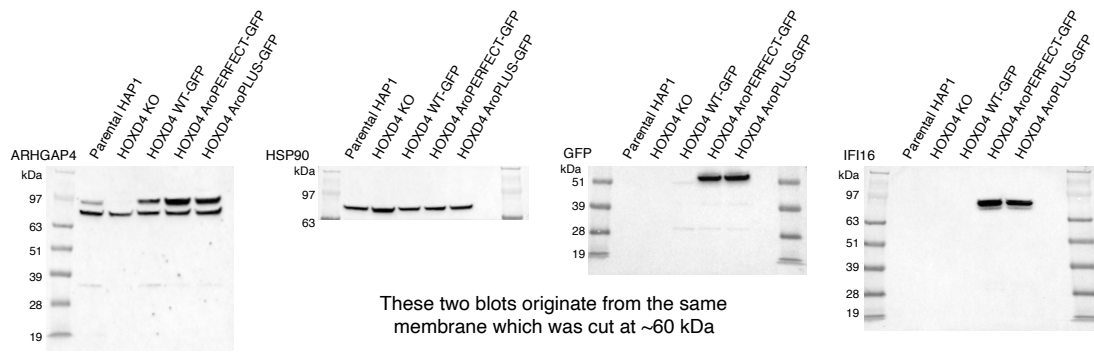

Rep 2

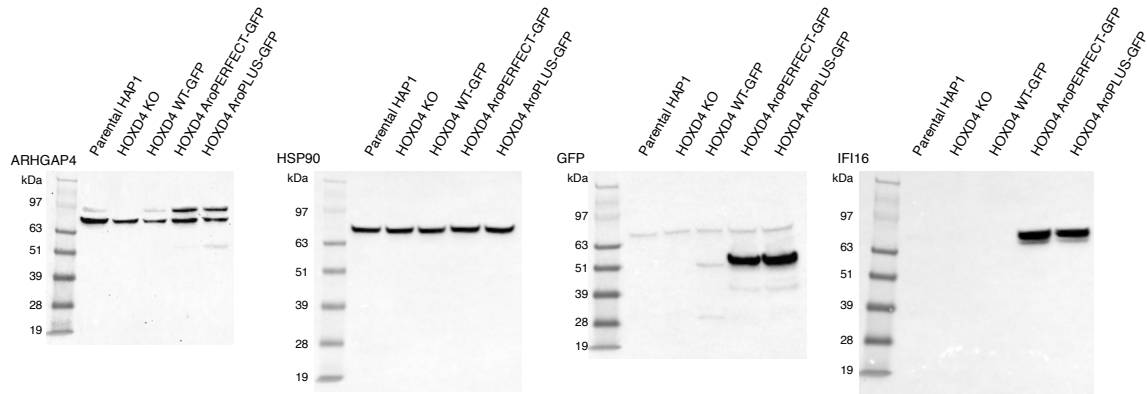

Supplement: Supplementary file 22 — Uncropped blot images for Fig. 3f. [file 41556_2024_1411_MOESM22_ESM.pdf]

Source Data Extended Data Figure 5

Uncropped blot images for Extended Data Figure 5c

Rep 1

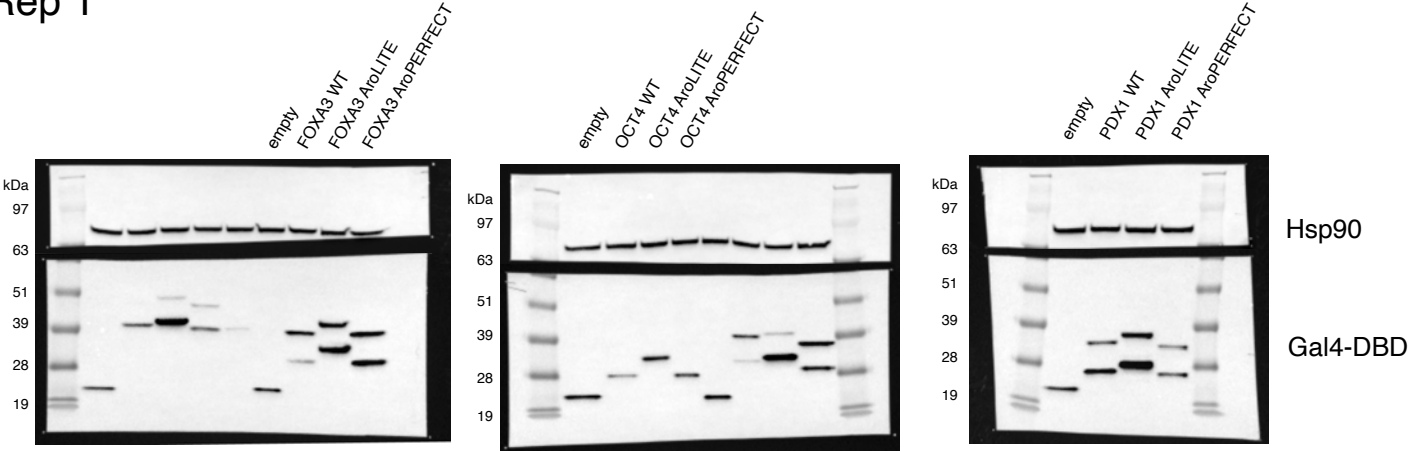

Rep 2

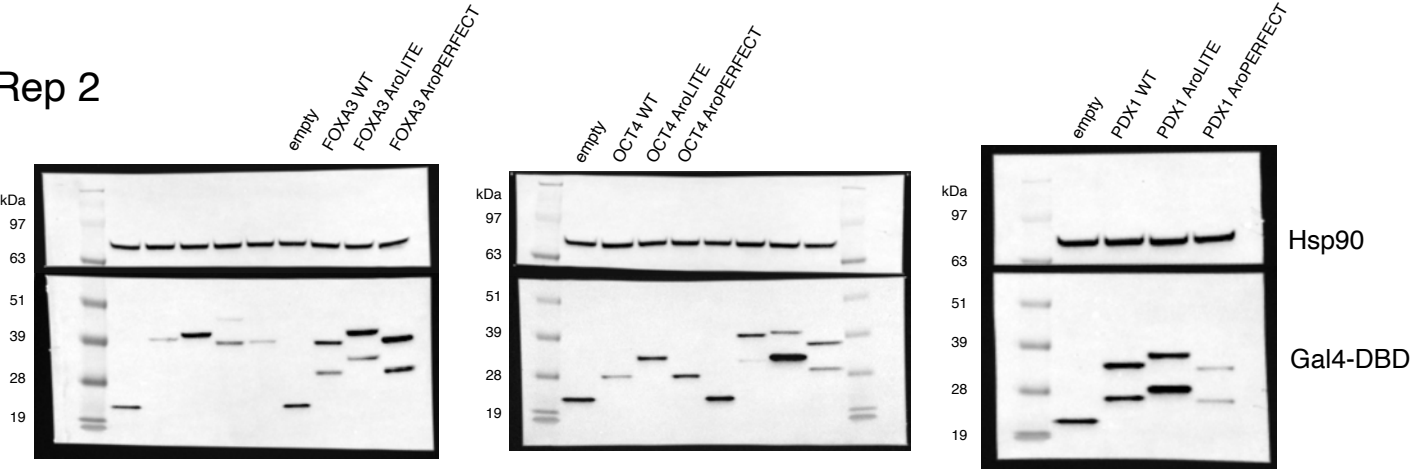

Supplement: Supplementary file 24 — Uncropped blot images for Extended Data Fig. 5c. [file 41556_2024_1411_MOESM24_ESM.pdf]

Source Data Extended Data Figure 7

Uncropped blot images for Extended Data Figure 7b

Rep 1

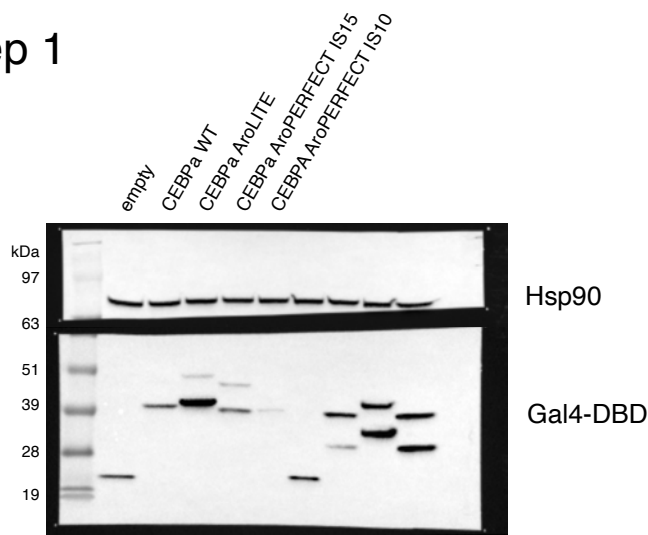

Rep 2

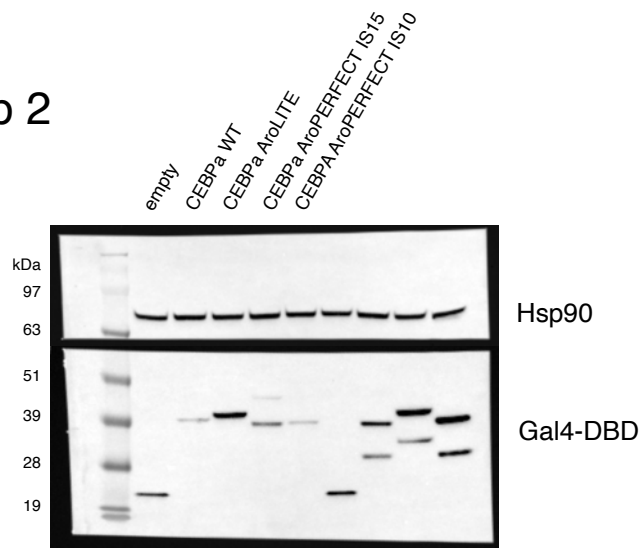

Supplement: Supplementary file 26 — Uncropped blot images for Extended Data Fig. 7b. [file 41556_2024_1411_MOESM26_ESM.pdf]

Source Data Extended Data Figure 10

Uncropped blot images for Extended Data Figure 10a

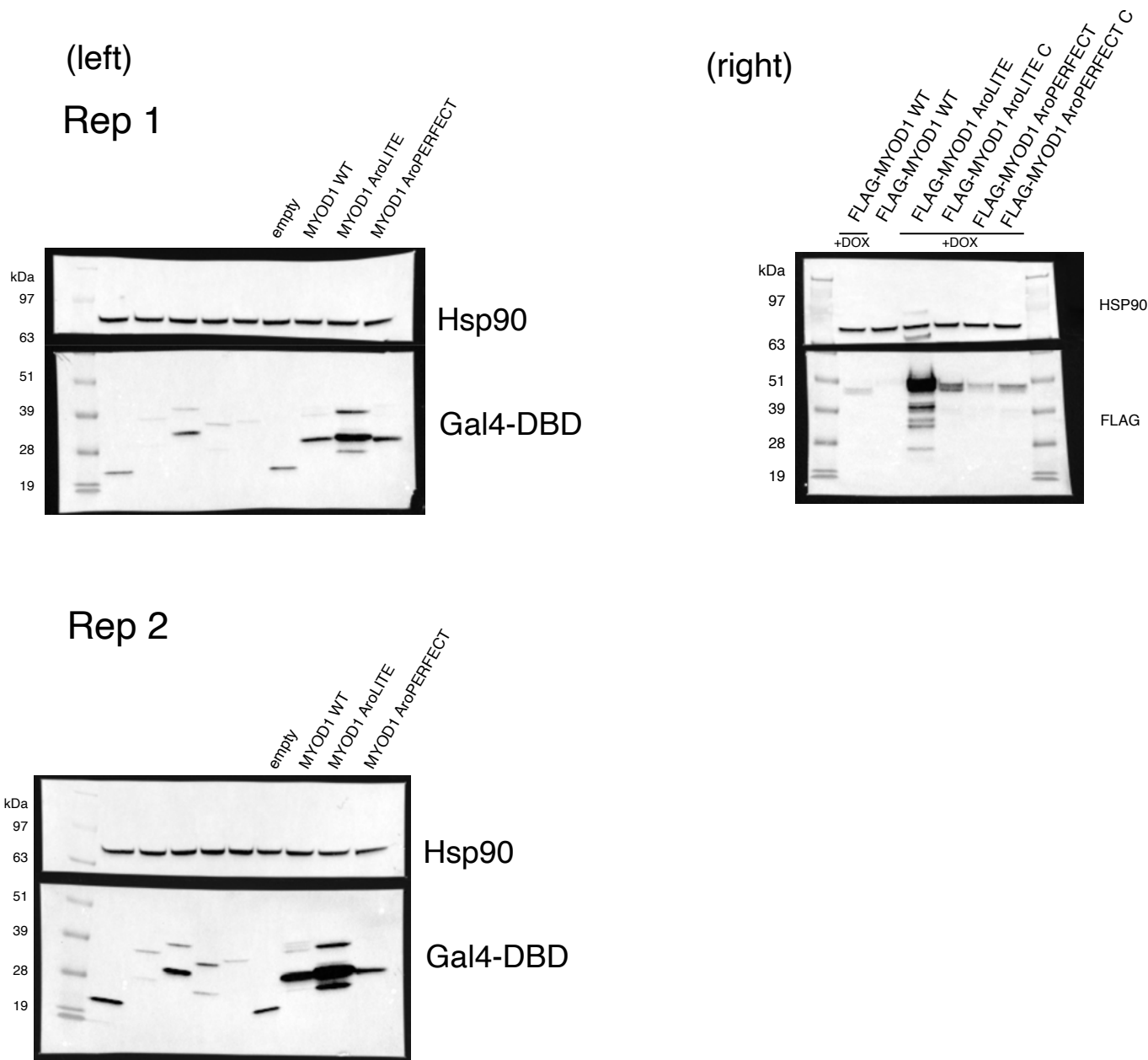

Supplement: Supplementary file 27 — Uncropped blot images for Extended Data Fig. 10a. [file 41556_2024_1411_MOESM27_ESM.pdf]
