# Supplementary material for: An activity-specificity trade-off encoded in human transcription factors
Source: Nat Cell Biol. 2024 Jul 5;26(8):1309–21. doi: 10.1038/s41556-024-01411-0 (PMC11321997; doi:10.1038/s41556-024-01411-0)

Source Extended Data Figure 4

Uncropped blot images for Extended Data Figure 4b

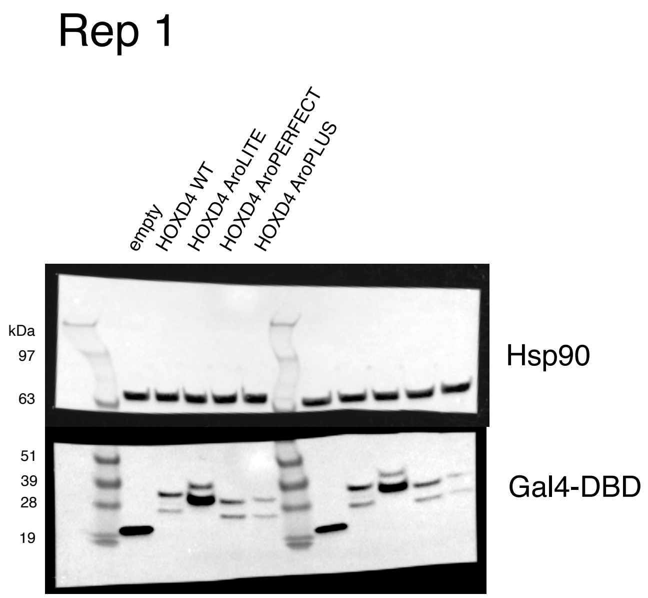

Uncropped blot images for Extended Data Figure 4g

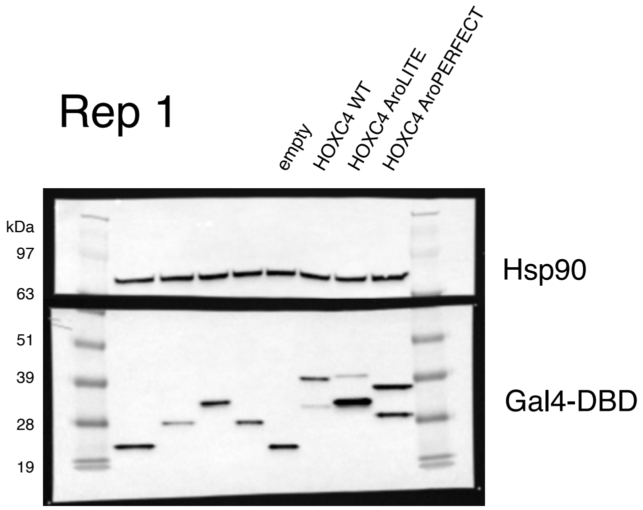

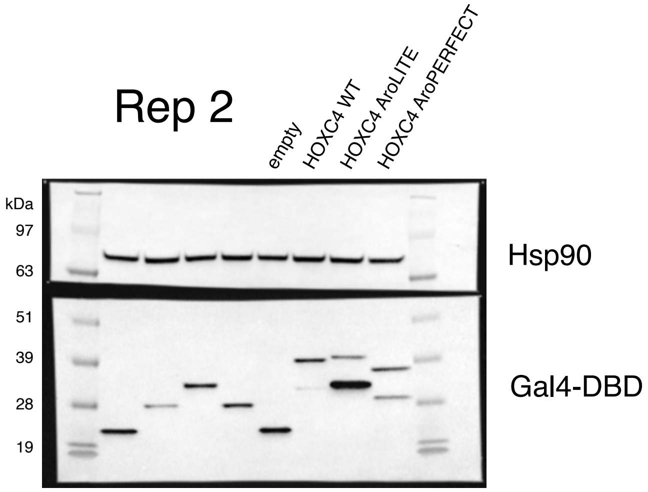

Supplement: Supplementary file 23 — Uncropped blot images for Extended Data Fig. 4b,g. [file 41556_2024_1411_MOESM23_ESM.pdf]
