# Supplementary material for: An activity-specificity trade-off encoded in human transcription factors
Source: Nat Cell Biol. 2024 Jul 5;26(8):1309–21. doi: 10.1038/s41556-024-01411-0 (PMC11321997; doi:10.1038/s41556-024-01411-0)

Source Data Extended Data Figure 6

Uncropped gel images for Extended Data Figure 6c

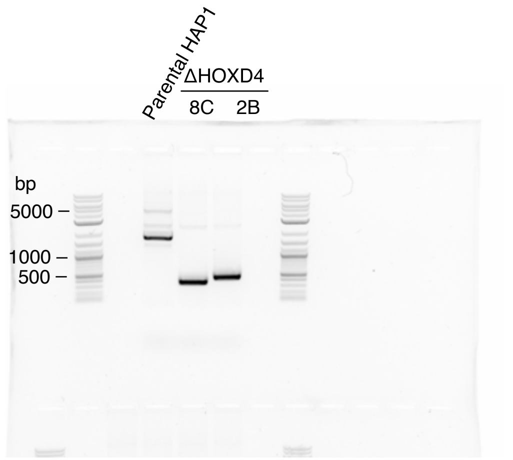

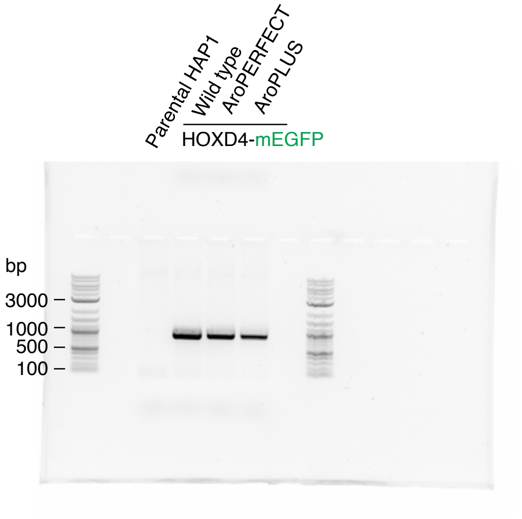

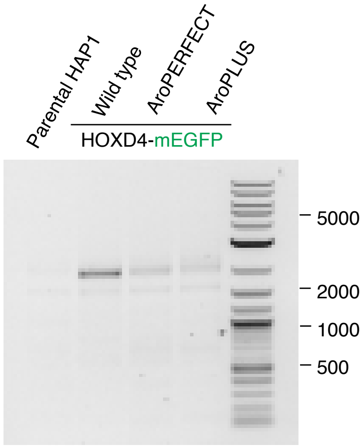

Uncropped blot images for Extended Data Figure 6f

(left)

Rep 1

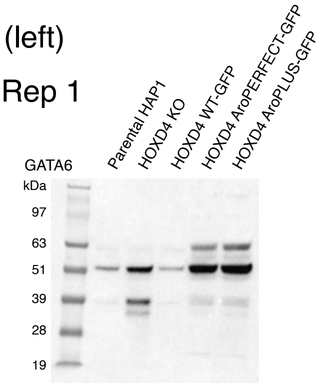

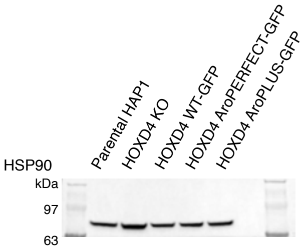

Rep 2

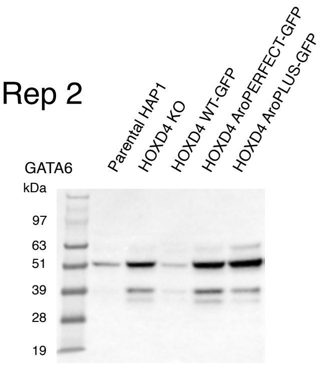

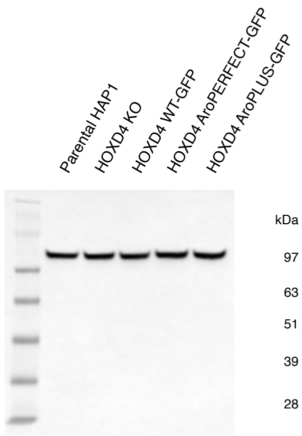

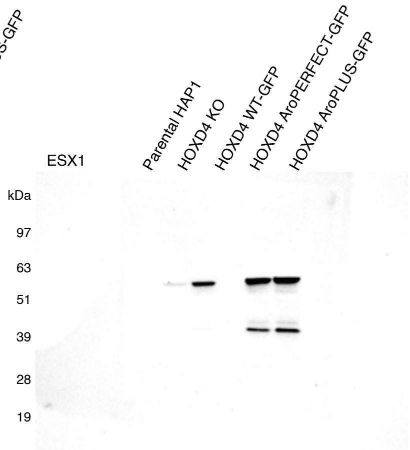

(right)

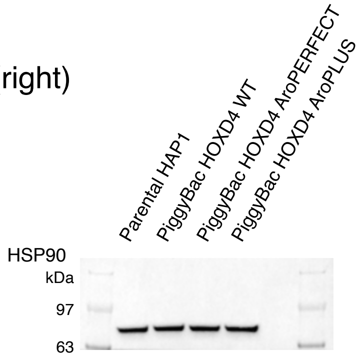

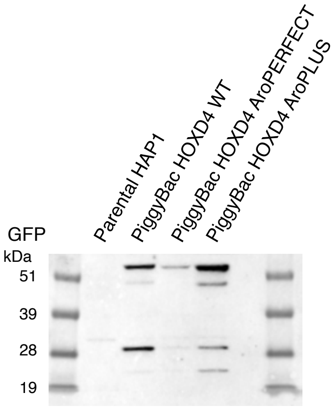

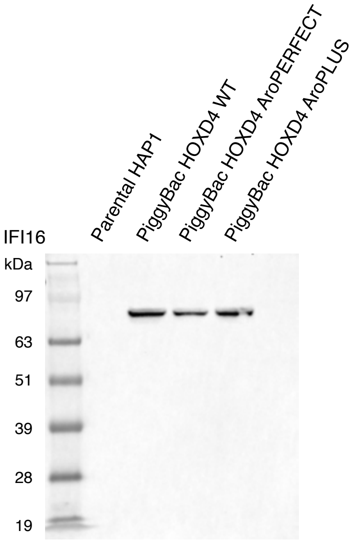

Supplement: Supplementary file 25 — Uncropped gel images for Extended Data Fig. 6c and uncropped blot images for Extended Data Fig. 6f. [file 41556_2024_1411_MOESM25_ESM.pdf]
